# Identification of the cell cycle characteristics of non-small cell lung cancer and its relationship with tumor immune microenvironment, cell death pathways, and metabolic reprogramming

**DOI:** 10.3389/fendo.2023.1147366

**Published:** 2023-04-06

**Authors:** Shengji Cao, Sitong Xiao, Jingyang Zhang, Shijun Li

**Affiliations:** ^1^ Department of Clinical Laboratory Medicine, First Affiliated Hospital of Dalian Medical University, Dalian, China; ^2^ Department of Clinical Laboratory Medicine, The Third People’s Hospital of Yuhang District, Hangzhou, China

**Keywords:** non-small cell lung cancer, cell cycle, pan-cancer analysis, tumor immune microenvironment, cell death pathways, metabolic reprogramming

## Abstract

**Background:**

The genes related to the cell cycle progression could be considered the key factors in human cancers. However, the genes involved in cell cycle regulation in non-small cell lung cancer (NSCLC) have not yet been reported. Therefore, it is necessary to evaluate the genes related to the cell cycle in all types of cancers, especially NSCLC.

**Methods:**

This study constituted the first pan-cancer landscape of cell cycle signaling. Cluster analysis based on cell cycle signaling was conducted to identify the potential molecular heterogeneity of NSCLC. Further, the discrepancies in the tumor immune microenvironment, metabolic remodeling, and cell death among the three clusters were investigated. Immunohistochemistry was performed to validate the protein levels of the ZWINT gene and examine its relationship with the clinical characteristics. Bioinformatics analyses and experimental validation of the ZWINT gene were also conducted.

**Results:**

First, pan-cancer analysis provided an overview of cell cycle signaling and highlighted its crucial role in cancer. A majority of cell cycle regulators play risk roles in lung adenocarcinoma (LUAD); however, some cell cycle genes play protective roles in lung squamous cell carcinoma (LUSC). Cluster analysis revealed three potential subtypes for patients with NSCLC. LUAD patients with high cell cycle activities were associated with worse prognosis; while, LUSC patients with high cell cycle activities were associated with a longer survival time. Moreover, the above three subtypes of NSCLC exhibited distinct immune microenvironments, metabolic remodeling, and cell death pathways. ZWINT, a member of the cell signaling pathway, was observed to be significantly associated with the prognosis of LUAD patients. A series of experiments verified the higher expression levels of ZWINT in NSCLC compared to those in paracancerous tissues. The activation of epithelial-mesenchymal transition (EMT) induced by ZWINT might be responsible for tumor progression.

**Conclusion:**

This study revealed the regulatory function of the cell cycle genes in NSCLC, and the molecular classification based on cell cycle-associated genes could evaluate the different prognoses of patients with NSCLC. ZWINT expression was found to be significantly upregulated in NSCLC tissues, which might promote tumor progression *via* activation of the EMT pathway.

## 1 Background

Lung cancer, a malignant tumor, is the most common type of cancer worldwide with the highest incidence and mortality (1). The histological types of lung cancer can be divided into small-cell lung cancer (SCLC) and non-small cell lung cancer (NSCLC) (2). Among them, the NSCLC accounts for about 80–85% of total lung cancer cases, and mainly includes lung adenocarcinoma (LUAD) and lung squamous cell carcinoma (LUSC) (3). Although there has been tremendous development in the clinical diagnosis and treatment of lung cancer, such as radiotherapy, chemotherapy, immunotherapy, molecular targeted therapy, etc., the 5‐year survival rate of NSCLC is still poor because the lung cancer is often insidious in the early stage and there are delays in the diagnosis (1, 4). Therefore, it is necessary to develop and investigate specific neoplasm markers for improving the prediction of the clinical outcomes and chemotherapy sensitivity of patients with NSCLC.

In the past years, a series of related breakthroughs have found the relationship between the regulation mechanism of the cell cycle and the development of a tumor. The major cause of tumorigenesis is the unrestricted cell proliferation after the cell cycle disorder and therefore, the tumor could be regarded as a cell cycle disease (5–7). It is well known that the driving mechanism and the regulatory mechanism of the cell cycle play important roles during cell proliferation. When the regulatory mechanism of the cell cycle is damaged, there can be uncontrolled cell growth, which may lead to the transformation of tumor cells. As there is a close association between the cell cycle and the tumor, the cell cycle could be considered one of the primary cellular mechanisms in the occurrence and development of cancer (8). Several studies have reported that therapy targeting cell cycle could serve as a reasonable treatment option to delay tumor progression by inhibiting tumor cell proliferation and inducing its apoptosis (6, 9, 10). As the vital genes related to the cell cycle might act as markers for pre-cancerous lesions or early-stage cancers, doctors could choose the best treatment for cancer patients to prolong their survival (11). Hence, it is necessary to investigate the key molecular signatures participating in cell cycle regulation in cancer cells. Furthermore, the cell cycle-related genes have not been found to predict the clinical outcomes and chemotherapeutic strategies in NSCLC patients. Therefore, the development of an NSCLC risk stratification tool and exploring the key gene from the cell cycle-related genes are important.

In this study, the roles of cell cycle-related genes in NSCLC were investigated by obtaining samples from the Cancer Genome Atlas (TCGA) database. Moreover, the cell cycle-related genes were acquired from the MsigDb platform. We identified 93 cell cycle-related genes that were associated with the tumor stage of NSCLC. This study comprehensively highlighted the genome and transcriptome characteristics of 93 genes in human tumors for the first time. Based on the cell cycle scores and cell cycle-related gene expression, we separated patients with NSCLC into three distinct types and evaluated their association with prognosis, metabolic reprogramming, immune microenvironments, and cell death pathways. Finally, we identified the hub gene ZWINT using bioinformatics. The association between ZWINT expression and patient prognosis, its potential role in tumor immunity, the clinical features of pan cancer, and the important pathways in cancer were determined using R.

## 2 Materials and methods

### 2.1 Sample collection and acquisition of genes associated with the cell cycle

The TCGA-LUAD and TCGA-LUSC cohorts were obtained from the TCGA GDC website and recognized as NSCLC cohorts. The data filtering and polishing were conducted using Perl and R programming (12). The TCGA-LUAD cohort consisted of 539 tumor samples and 59 paracancerous samples, while the TCGA-LUSC cohort consisted of 502 tumor samples and 49 paracancerous samples. All these RNA-seq data were initially converted to log format and then the sva package was applied to complete the bulk rectification procedure. The clinical information about each patient was also collected and compiled. Finally, the cell cycle-related dataset was obtained and downloaded from the MsigDb platform and the “REACTOME CELL CYCLE” dataset was compiled (13, 14). A total of 693 genes were present in this dataset, all of which were considered to be related to the cell cycle.

### 2.2 Identification of the cell cycle genes associated with the development of NSCLC

The conversion of normal lung epithelial cells to NSCLC cells is considered a typical example of carcinogenesis. Therefore, a significant role played by genes in carcinogenesis has been a topic worthy of scientific investigation. The cell cycle genes associated with the development of NSCLC were identified by comparing the malignant and noncancerous tissues from the TCGA-LUAD and TCGA-LUSC cohorts. The cutoff values for differential expression analysis were set as follows: | logFC | > 1.5 and FDR < 0.05. Finally, the findings from the TCGA-LUAD and TCGA-LUSC cohorts were intersected to provide a list of differentially expressed genes.

The patients with NSCLC who acquire a tumor eventually progress from stage I to stage IV. Therefore, it is crucial to determine the cell cycle genes that are associated with the clinical stage of NSCLC patients. The GEPIA2 platform developed by Peking University was applied to facilitate the analysis (15, 16). The specific parameters used were as follows: “Expression DIY” toolbar, “Stage Plot” interface, and “LUAD + LUSC” dataset. Only the cell cycle genes with a P value of less than 0.05 were selected and further evaluated using bioinformatics.

### 2.3 Pan-cancer analysis

The pan-cancer cohort of TCGA was downloaded and integrated to analyze the involvement of the aforementioned cell cycle-related genes in diverse human cancers. The gene expression, prognostic value, mutation type, methylation level, and pathway regulation, among other factors, were reviewed by referring to the previously published pan-cancer analysis methods (17–19).

The differential expression analyses of cancer and paracancerous tissues at the pan-cancer level were conducted using R software packages, such as ggplot2, randomcoloR, ggpubr, GSVA, clusterProfiler, impute, and ComplexHeatmap, and the findings were represented as a heat map (20–22). The color of the dot on the heat map indicated whether the gene was up-regulated or down-regulated in the cancer tissue, and the size of the dot indicated the statistical P value. The greater the size of the dot, the greater the statistical significance of the finding.

The pan-cancer prognostic characteristics of the aforementioned cell cycle-related genes were studied in detail using the R packages survival and pheatmap, and a heat map was constructed. Red represents a dangerous gene, indicating that the greater the level of gene expression, the worse the prognosis of patients; blue shows a protective gene indicating that the higher the level of gene expression, the better the prognosis of patients. Gray suggests that the gene has no predictive association in patients with this form of malignancy.

A series of R packages, such as ggplot2, randomcoloR, tidyverse, magrittr, readxl, stringr, maftools, dplyr, reshape2, and RColorBrewer were used for single nucleotide variant (SNV) and copy number variant (CNV) analyses. The frequency of SNV mutations in each gene in a tumor is represented as a heat map, and the type of mutation of each SNV is represented as a waterfall map. Each hue in the CNV bar chart reflects a distinct type of tumor.

The pan-cancer methylation levels of the cell cycle genes were summarized using the R packages ggplot2, ChAMP, randomcoloR, ggpubr, GSVA, clusterProfiler, impute, and ComplexHeatmap. A red dot indicates a high amount of methylation of the gene in this type of tumor, whereas the blue dot indicates a low level of methylation. The size of the dot shows the P value; therefore, the bigger the dot, the greater the statistical significance.

A comprehensive analysis of the association between the cell cycle pathways and other traditional tumor pathways was conducted using clusterProfiler, limma, ggplot2, ggpubr, GSVA, and other R packages. Specifically, the relative score of each pathway was calculated using the GSVA package to indicate the relative activity of the pathway, and the correlation values between the cell cycle pathway and other pathways were examined by the correlation test, before being represented as a heat map.

### 2.4 Cluster analysis

First, univariate Cox regression analysis of the TCGA-LUAD cohort was conducted to determine the cell cycle-related genes with prognostic values, which were further used for conducting cluster analysis. For both the TCGA-LUAD and TCGA-LUSC cohorts, GSVA methods were used for calculating the cell cycle score of each sample (23, 24). We performed cluster analysis based on the expression levels of the samples using ward.D. Before classifying the tumor tissues into three subtypes based on the distinct cell cycle activities, we evaluated the mRNA expression levels in normal tissues. The survival and survminer programs were used to evaluate the prognosis of patients with different subtypes of NSCLC to highlight the clinical value of cluster analysis.

### 2.5 Analysis of tumor metabolic reprogramming, immune microenvironment, and cell death status

42 conventional metabolic pathways, 33 immune-related pathways, and 10 cell death pathways were classified using the MsigDb platform. The metabolic score, immunological score, and cell death score for each NSCLC sample were determined using the GSVA program. The Kruskal test was used to assess the pathway activity between the three subtypes of the cell cycle. Moreover, a comprehensive analysis of the differences in immune cell infiltration and gene expression at immune checkpoints was conducted to exhaustively describe the changes in the immune milieu across subtypes. The TIMER2.0 platform offers several immunological algorithms, including TIMER, CIBERSOFT, QUANTISEQ, EPIC, etc. (25, 26). The Kruskal test was used to assess the differences in the immune cell infiltration and gene expression at the immunological checkpoints across subtypes, and only the findings with P < 0.05 were represented.

### 2.6 Biological functional analysis of the ZWINT gene

The expression patterns of the ZWINT gene in both healthy and malignant tissues were obtained from Genotype-Tissue Expression (GTEx), Cancer Cell Line Encyclopedia (CCLE), and The Cancer Genome Atlas (TCGA) (27, 28). The information on ZWINT mRNA expression in normal tissues was obtained from GTEx, a dataset containing expression data of 31 healthy tissues, while the information on the expression distribution across various cancer cell lines was obtained from CCLE, a database containing information on more than 1100 cancer cell lines. TCGA provided information on the differential expression of genes between malignant and noncancerous tissues. The GTEx and TCGA databanks were accessed using the UCSC Xena platform.

Further, the linkages between ZWINT expression and clinical outcomes were obtained using the information on patient survival from the TCGA database. Disease-free interval (DFI), disease-specific survival (DSS), overall survival (OS), and progression-free interval (PFI) were used to evaluate the correlation between mRNA expression levels and patient survival rates (29). ZWINT expression and survival outcomes were analyzed using Kaplan-Meier (KM) and Cox analyses for patients with different types of cancer. KM curves and forest plots were created in R. Then, a correlation analysis of clinicopathological data such as tumor grade, tumor stage, gender, age, race, and tumor status was conducted using the “limma” and “ggpubr” packages in R (30). Both the “survminer” and “survival” packages in R were used to generate the survival curves. A P value of 0.05 was considered as the threshold of statistical significance.

The Tumor Immune Estimation Resource 2.0 online server is a useful resource for systematically analyzing the immune infiltrates across different cancer types. Initially, it was used to examine the differences in ZWINT expression between the tumor and normal tissues in all the TCGA cohorts. A correlation analysis between ZWINT expression and immune infiltration was conducted using several immunological deconvolution techniques. More importantly, the correlation between ZWINT expression and immune checkpoint levels was also evaluated in this study.

Biomarker Exploration of Solid Tumors website is a publicly free web-based platform for omics data. It was used to further explore the contribution of ZWINT in NSCLC. The GO and KEGG analyses for ZWINT were based on the TCGA-LUAD and TCGA-LUSC cohorts. Based on the median value of ZWINT expression, LUAD, and LUSC patients were stratified into high-ZWINT and low-ZWINT subgroups. Then, the mutation profiles between high-ZWINT and low-ZWINT subgroups were intensively studied. Besides, the predictive ability of ZWINT in the immunotherapy of NSCLC patients was also analyzed. Finally, the expression levels of the ZWINT gene in different clinical subgroups of NSCLC patients were comprehensively investigated.

To highlight the important roles of the ZWINT gene in tumor immune microenvironment, metabolic remodeling, and cell death, GSEA analyses were conducted in pan-cancer tissues following the previously described methods. Based on the expression levels of the ZWINT gene in pan-cancer tissues, it was ranked from high to low. The first 30% of the tumor samples were considered as ZWINT high expression subtype, and the last 30% of the tumor samples were regarded as ZWINT low expression subtype. Further, the tumor samples of different subtypes were analyzed by GSEA.

### 2.7 Clinical sample collection

We collected 30 NSCLC tissues and 30 paired paracancerous tissues from our hospital from 2021-08 to 2021-10. The tissues were immediately frozen in liquid nitrogen after surgically resecting specimens to extract total RNA. Moreover, 50 pairs of paraffin-embedded pathological specimens obtained from 2015-12 to 2016-12 were collected in this study. The essential information and clinically-relevant pathological information of patients with NSCLC are shown in **Tables 1**, **2**, respectively. All the patients with NSCLC from whom the tissues were collected were pathologically confirmed to have NSCLC and were treated in the First Affiliated Hospital of Dalian Medical University. The informed consent was provided by the First Affiliated Hospital of Dalian Medical University. All the patients with NSCLC refused chemotherapy and radiation treatment before surgery. The Ethics Committee of the First Affiliated Hospital of Dalian Medical University approved this study.

**Table 1:** Basic information of the NSCLC patients.

Basic information	Gender		Age	
	Male	Female	≥60	<60
Number	26	24	30	20

**Table 2:** Clinically-relevant pathological information.

Pathological information	Histological type		Differentiation		Stage		Lymphatic metastasis	
	Squamous	Adenocarcinoma	High Middle	Minor	I + II	III + IV	Yes	No
Number	18	32	38	12	35	15	24	26

### 2.8 Isolation of the total RNA and validation of the expression level of the ZWINT gene by quantitative real-time PCR

Total RNA was extracted from the human NSCLC tissues by the RNAex Pro RNA reagent based on the manufacturer’s instructions. Subsequently, the total RNA was reverse-transcribed using the Evo M-MLV RT Kit with gDNA Clean. The expression level of the ZWINT gene was calculated by RT-PCR using SYBR^®^ Green Premix Pro Taq HS qPCR Kit. All the reagents above were procured from Accurate Biology. The ZWINT gene was normalized to β-actin. The ΔΔCt method was used for quantifying the level of RNA expression. The primer sequences for ZWINT and β-actin were as follows:

ZWINT, 5’-AGGAGGACACTGCTAAGGG-3’(Forward), 5’- AGGTGGCCTTCAGCTCTTTC-3’ (Reverse); β-actin, 5’-CATGTACGTTGCTATCCAGGC-3’ (Forward), 5’- CTCCTTAATGTCACGGACGAT -3’ (Reverse).

### 2.9 Immunohistochemical staining

The protein expression of ZWINT was tested by immunohistochemical staining (IHC). The paraffin-embedded tissue slides were immune stained with anti-N-cadherin, anti-E-cadherin, vimentin, and ZWINT. After dewaxing, hydration, and epitope extraction, the sections were placed in 3% hydrogen peroxide for 15 min to inhibit endogenous peroxidase activity. Subsequently, the sections were incubated overnight with a solution containing the appropriate primary antibody. Then, 50 µL of secondary antibody was added in a sequence and incubated at room temperature for 20 min. IHC staining was carried out according to the manufacturer’s protocol. The results were blindly assessed independently by two pathologists. Positive ZWINT-staining rate was considered on a scale of 0 to 4, with 1 indicating (0–25%), 2 (26–50%), 3 (51–75%), and 4 (76–100%). Staining intensity was rated as follows: 0 (no staining), 1 (weak staining), 2 (moderate staining), or 3 (strong staining). IHC score was calculated as a product of positive staining rate and intensity score. The patients were divided into high and low-expression groups, with a score of 2 or less considered as low expression, and a score of more than 2 considered as high expression.

## 3 Results

### 3.1 Identification of the cell cycle genes associated with the occurrence and development of NSCLC

A total of 148 genes involved in the cell cycle were determined to be differently expressed in LUAD and paracancerous tissues (**Supplementary Table 1**). 210 cell cycle-related genes were identified to be differently expressed in malignant and noncancerous LUSC tissues (**Supplementary Table 2**). The intersection of the LUAD and LUSC results revealed 143 potential cell cycle-related genes strongly linked with NSCLC (**Supplementary Figure 1**). There is a clinical progression of NSCLC as the tumor grows (StageI-StageII-StageIII-StageIV). Further, the cell cycle genes associated with the stage of NSCLC were identified. The findings from this study indicated that 93 out of 143 potential genes were linked with the tumor stage (**Supplementary Table 3**). Therefore, these 93 genes were retained and used for further analysis.

### 3.2 Pan-cancer characterization of the 93 cell cycle genes

**Figure 1:**
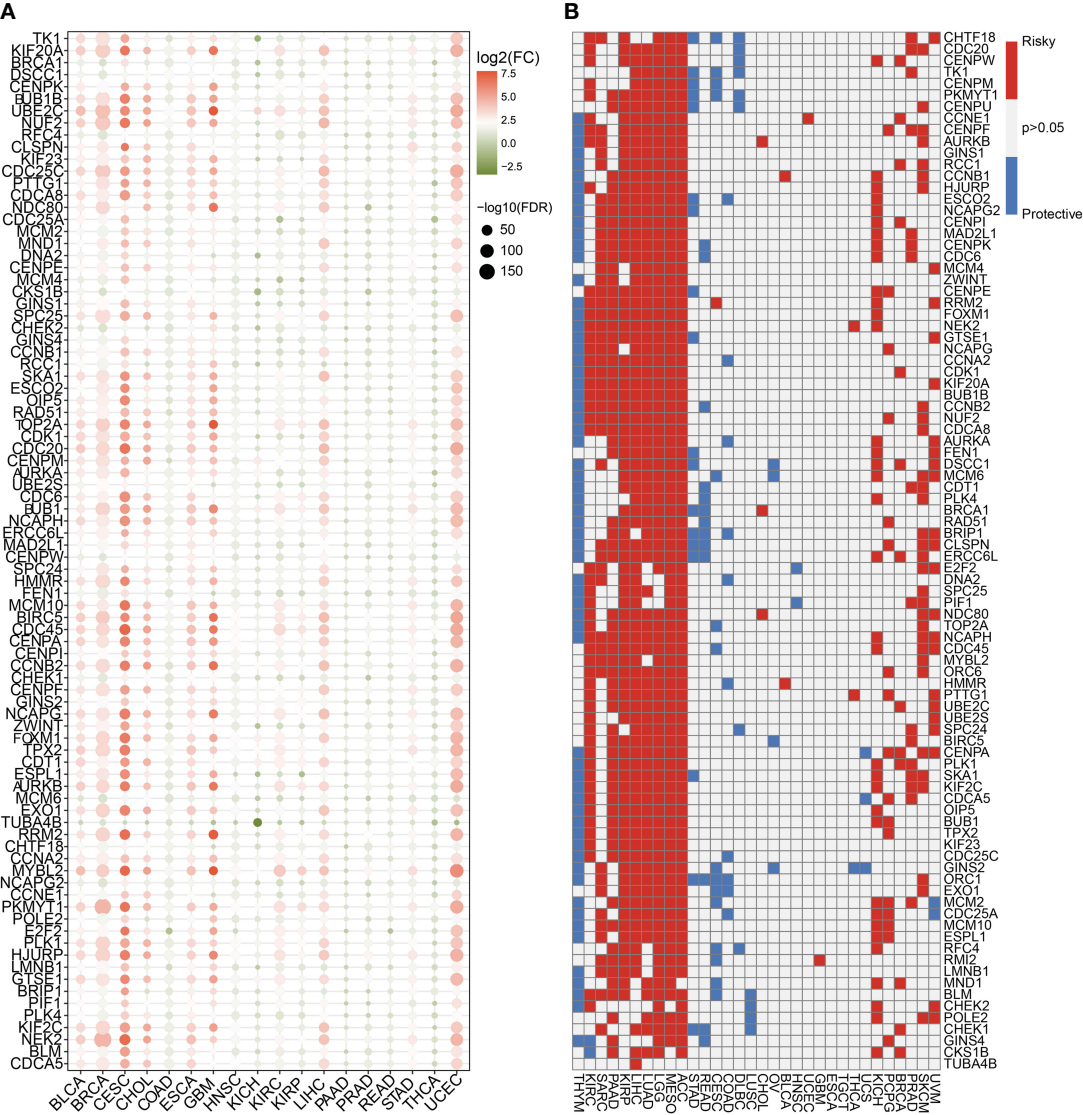
Expression traits and prognostic values of the cell cycle-related genes in pan cancer. **(A)** mRNA expression levels of the 93 cell cycle-related genes in other human tumors (P < 0.05). **(B)** Clinical outcomes of the cell cycle in pan cancer. White color (P > 0.05) indicates no statistical difference. Red color indicates the risk factor, while the blue color indicates the protective factor.

**Figure 2:**
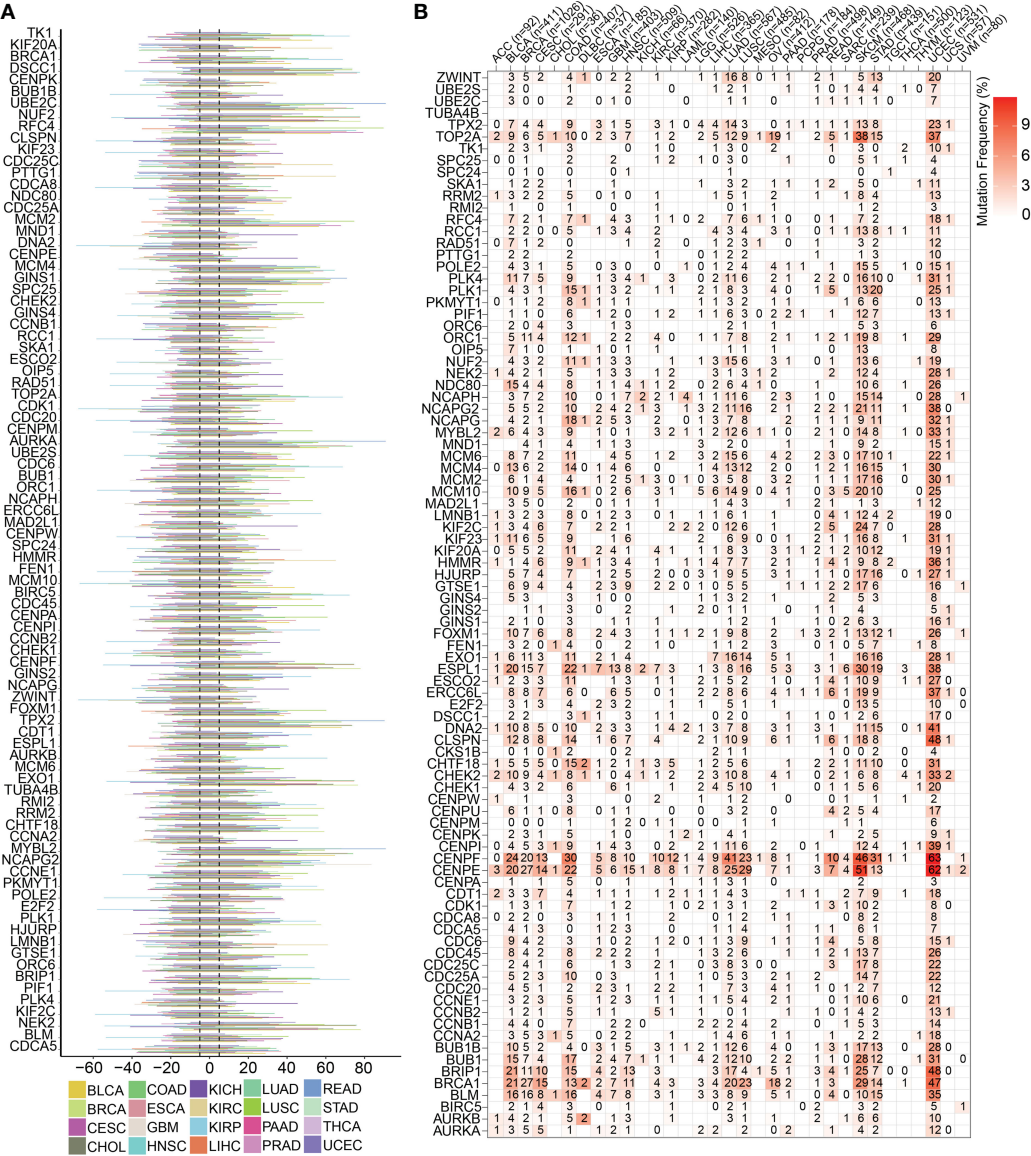
Genomics traits of cell cycle-related genes in pan cancer. **(A)** CNV outcomes of the 93 cell cycle-related genes in different types of cancers. The length line represents the wave frequency of the cell cycle-related genes in human malignant tumors. **(B)** Heatmap representing the SNV mutations for the 93 cell cycle-related genes.

**Figure 3:**
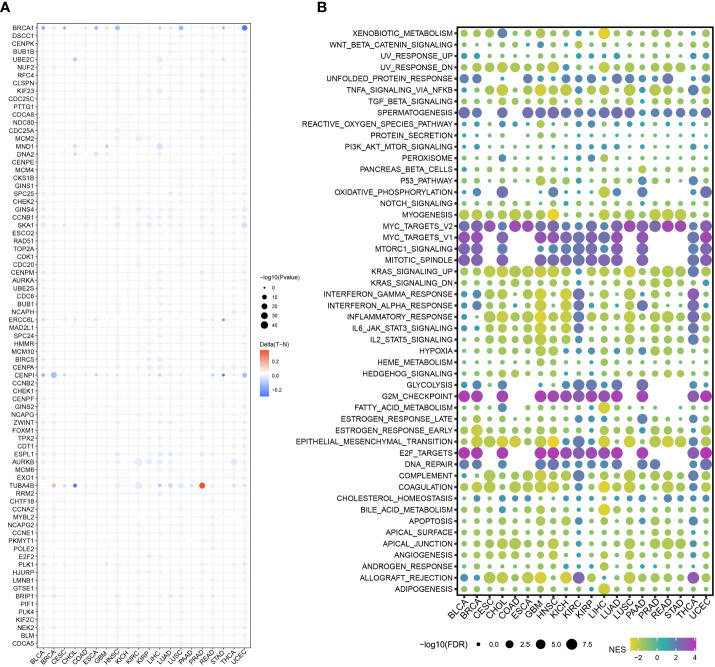
Methylation levels and pathway correlation of the cell cycle-related genes in pan cancer. **(A)** DNA methylation of the 93 cell cycle-related genes in different types of cancers (red to blue represents high to low). **(B)** 93 cell cycle-related genes were correlated with several classical tumor-related pathways (red to blue represents high to low).

To highlight the significance of the 93 genes in carcinogenesis and tumor development, a pan-cancer investigation was performed to exhaustively characterize their genomic and transcriptome properties in different human cancers. Except for NSCLC, practically all the genes showed highly up-regulated expression in the cancerous tissues of CESC, CHOL, GBM, LIHC, and UCEC (**Figure 1A**). However, a downregulated expression trend was observed in the cancer tissues of COAD and THCA (**Figure 1A**). This not only confirms the heterogeneity among tumors but also indicates that these genes may play different roles in different types of tumors. Importantly, our data indicated that a majority of these 93 genes perform deleterious functions in KIRC, SARC, PAAD, KIRP, LIHC, LUAD, LGG, MESO, ACC, KICH, PCPG, BRCA, PRAD, SKCM, and UVM (**Figure 1B**). In other words, as the expression of these genes increases, there is a clinical worsening in patients with these types of malignancies. For such cancer patients, gene-targeting techniques may provide a novel therapeutic option. However, these genes have a protective function in other types of cancer, including THYM, STAD, READ, COAD, DLBC, and LUSC (**Figure 1B**). Moreover, we also visualized their genetic characteristics. **Figure 2A** shows the CNVs. In individuals with distinct types of malignancies, several genes involved in the cell cycle exhibited considerably distinct genomic features, particularly CNV levels. The SNV mutations were more prevalent in patients with BLCA, BRCA, CESC, COAD, LUAD, LUSC, SKCM, STAD, and UCEC tumors (**Figure 2B**). It was evident that the degree of methylation of genes might impact the level of gene expression. Therefore, the methylation levels of these genes were examined in several tumor types. The data indicated that the degree of methylation in cancerous tissues is often lower than that in the surrounding tissues (**Figure 3A**). In addition, pathway enrichment analysis revealed that these cell cycle genes were significantly correlated with several classical tumor-related pathways, indicating that the cell cycle is intrinsically linked to tumor immune microenvironment, metabolic reprogramming, cell death, angiogenesis, and other biological phenomena (**Figure 3B**).

### 3.3 Cluster analysis of NSCLC patients based on the cell cycle gene expression characteristics and pathway activity

**Figure 4:**
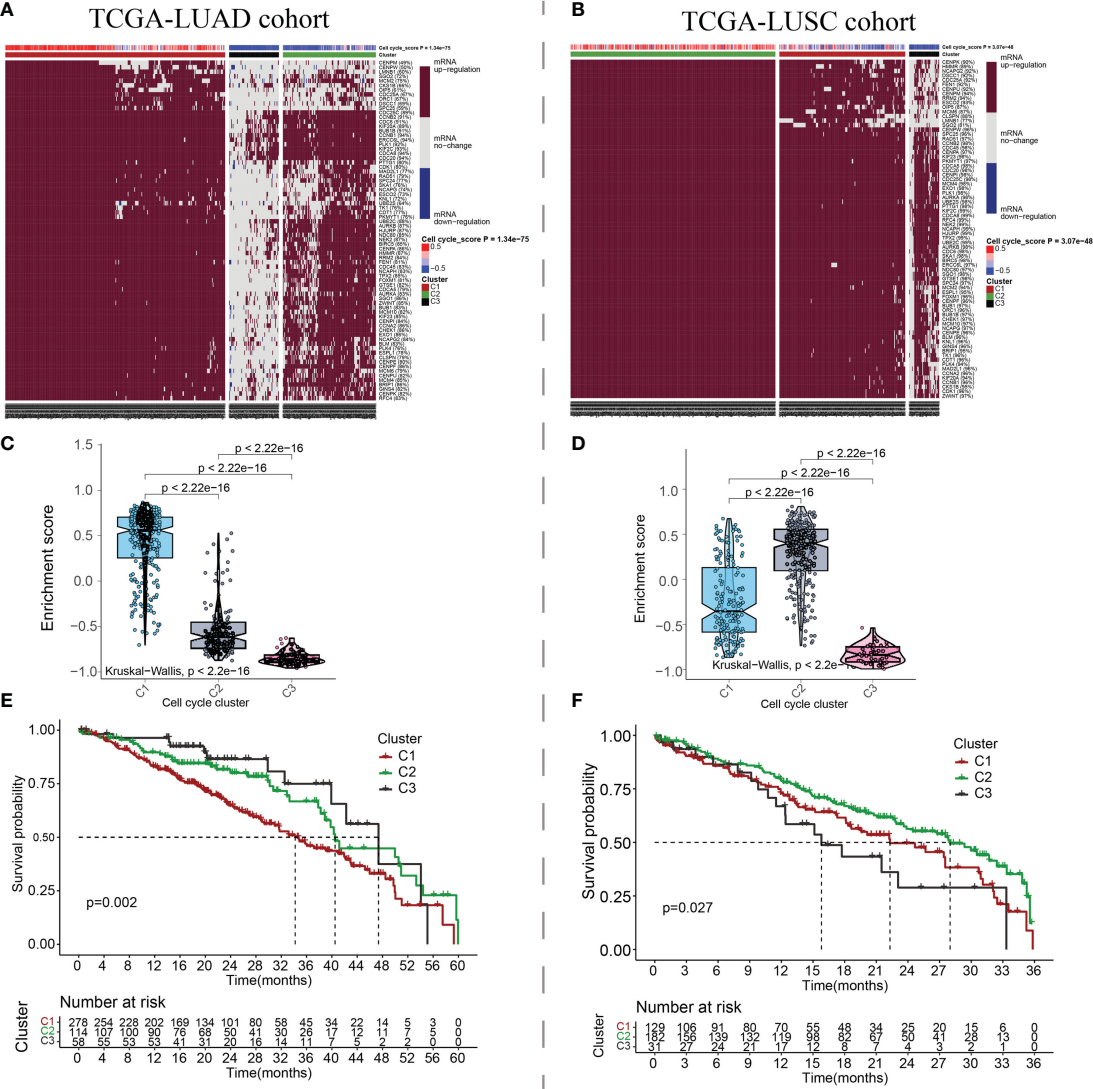
Cluster analysis based on the cell cycle-related genes. The patients with NSCLC in the TCGA-LUAD and TCGA-LUSC cohorts were successfully grouped into 3 clusters for LUAD **(A)** and LUSC **(B)**. Pathway enrichment scores followed the trend C1 > C2 > C3 in LUAD **(C)** and C2 > C1 > C3 in LUSC **(D)**. Three different clusters showed different survival curves. Cluster 1 has the worse survival rate in LUAD **(E)**. However, Cluster 1 has the worse survival rate in LUSC **(F)**. x indicates survival time and y indicates survival rate.

**Figure 5:**
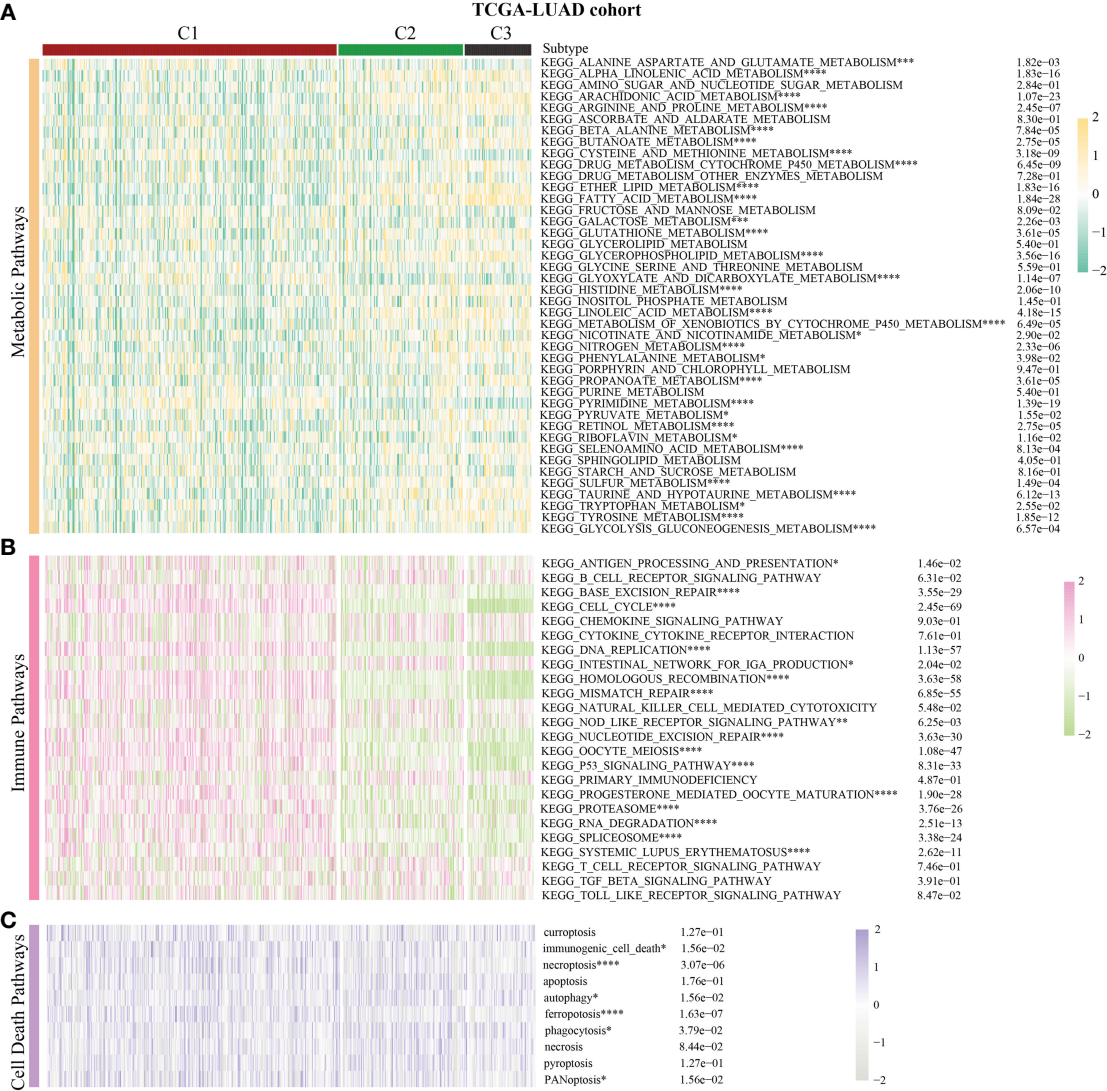
Correlation of the cell cycle-related gene scores with metabolic reprogramming, immunological microenvironment, and cell death pathways in LUAD. **(A)** Activity of well-recognized metabolic reprogramming in the three clusters for LUAD. **(B)** Activity of well-recognized immune pathways in the three clusters for LUAD. **(C)** Correlation between the cell cycle-related gene scores and cell death pathways for LUAD. * indicates p < 0.05; ** indicates p < 0.01; *** indicates p < 0.001; and **** indicates p < 0.0001.

**Figure 6:**
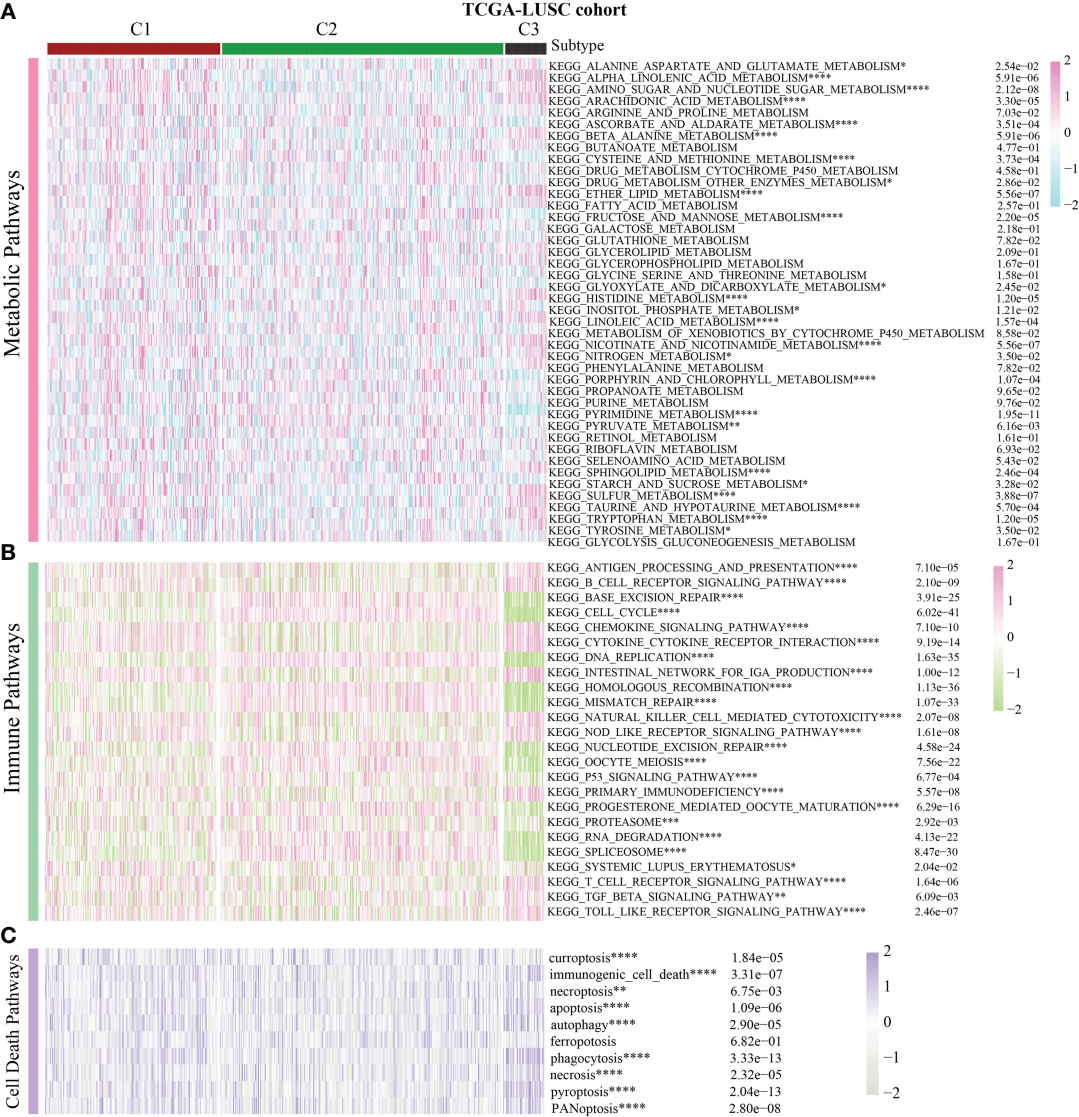
Correlation of the cell cycle-related gene scores with metabolic reprogramming, immunological microenvironment, and cell death pathways in LUSC. **(A)** Activity of well-recognized metabolic reprogramming in the three clusters for LUSC. **(B)** Activity of well-recognized immune pathways in the three clusters for LUSC. **(C)** Correlation between the cell cycle-related gene scores and cell death pathways for LUSC. * indicates p < 0.05; ** indicates p < 0.01; *** indicates p < 0.001; and **** indicates p < 0.0001.

Previous pan-cancer research findings revealed that the cell cycle influences LUAD and LUSC differently or possibly contrary to each other (**Figure 1B**). To further elucidate the underlying molecular heterogeneity of NSCLC patients, a cluster analysis was performed followed by pattern characterization. First, a univariate COX regression analysis was conducted to identify 75 genes associated with prognosis. The identification of these prognostic genes can be useful in distinguishing clinically meaningful molecular subtypes more effectively (**Supplementary Table 4**). The clustering results are shown in **Figures 4A, B**. All the cancer patients, both from the TCGA-LUAD and TCGA-LUSC cohorts, were successfully classified into three subtypes (C1, C2, and C3). In the TCGA-LUAD cohort, the trend of pathway enrichment score was C1 > C2 > C3, but in the TCGA-LUSC cohort, the pathway enrichment score sequence was C2 > C1 > C3 (**Figures 4C, D**). Interestingly, in the TCGA-LUAD cohort, the survival times of patients followed the trend C3 > C2 > C1, but in the TCGA-LUSC cohort, the trend was C2 > C1 > C3 (**Figures 4E, F**). A comprehensive examination of metabolic reprogramming, immunological microenvironment, and cell death pathways was performed to completely identify the intrinsic molecular properties of several subtypes of the cell cycle (**Figures 5**, **6**). The patients with LUAD and LUSC exhibited three fundamental metabolic abnormalities associated with metabolic reprogramming with a shift in their cell cycle activity, such as alanine aspartate and glutamate metabolism, alpha-linolenic acid metabolism, arachidonic acid metabolism, ether lipid metabolism, histidine metabolism, nitrogen metabolism, glyoxylate and dicarboxylate metabolism, and sulfur metabolism (**Figures 5A**, **6A**). Innate immune response and adaptive immune response exhibited notable differences among cell cycle subtypes, such as antigen processing and presentation (**Figures 5B**, **6B**). In addition, several subtypes of the cell cycle were followed by distinct cell death mechanisms, such as immunogenic cell death, necroptosis, phagocytosis, and PANoptosis (**Figures 5C**, **6C**).

### 3.4 Biological analysis of the ZWINT gene

**Figure 7:**
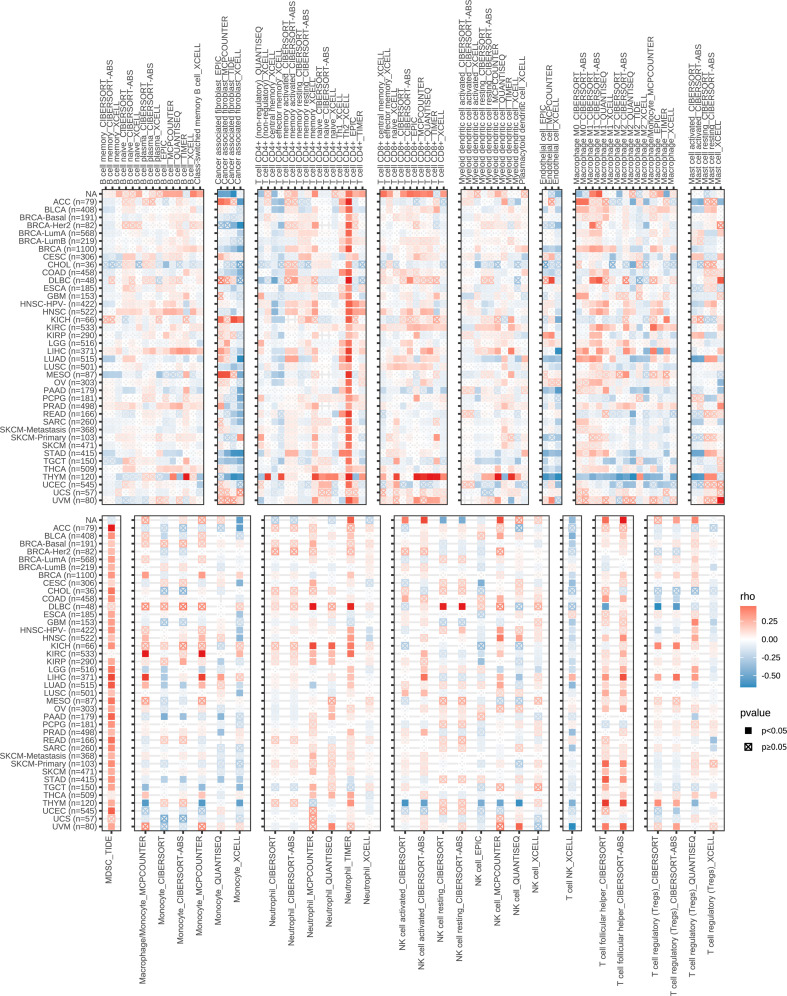
Correlation between the expression levels of ZWINT gene and immune cell infiltration in pan cancer. (Red to blue color represents high to low expression levels).

As a traditional cell cycle-related gene, the ZWINT gene has been identified to regulate the onset and development of several types of cancers; however, its association with NSCLC remains unknown. Therefore, the expression of the ZWINT gene in pan cancer and the possible functions of NSCLC were examined. Combining the histology data from TCGA and GTEx established that ZWINT was significantly overexpressed in several types of human cancers, indicating its significant role in carcinogenesis (**Supplementary Figures 2A, B**). Comprehensive pan-cancer investigation based on the univariate COX regression analysis and KM analysis indicated the prognostic significance of the ZWINT gene (**Supplementary Figures 3**–**7**). Immune correlation research revealed a significant relationship between the ZWINT gene and infiltrating immune cells, such as B cells, CD4+ T cells, cancer-associated fibroblasts, CD8+ T cells, macrophages, neutrophils, and NK cells (**Figure 7**). Immune checkpoints are the primary limiting criteria for the activity of immunological cells. As shown in **Supplementary Figure 8**, we also studied the influence of the ZWINT gene on the expression levels of different types of immune checkpoints. Immunotherapy targeting immune checkpoints is an emerging research area in cancer treatment, and the study results indicated that the ZWINT gene can be used as a predictor of immunotherapy response to a certain extent (**Supplementary Figure 9**).

**Figure 8:**
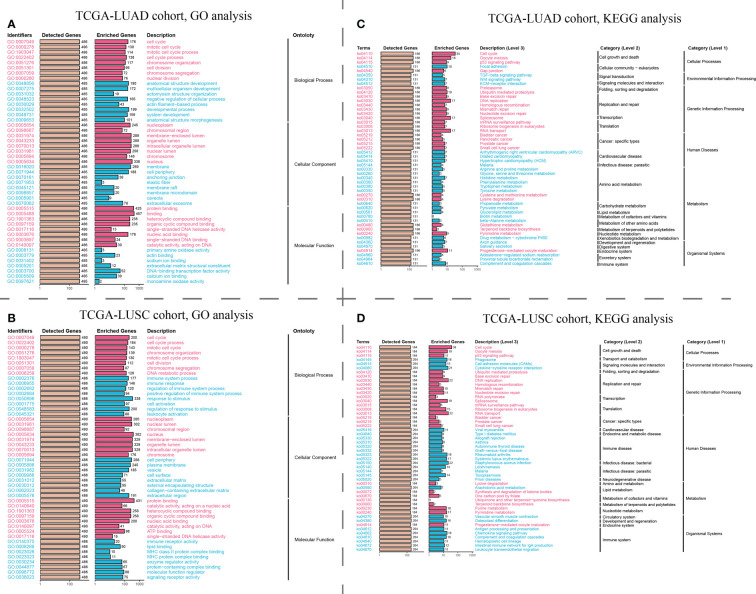
Pathway enrichment analysis of the hub ZWINT gene by GO and KEGG in LUAD and LUSU. **(A, B)** GO and KEGG analysis for investigating the relationship between the classic cancer pathways and the hub ZWINT gene in LUAD. **(C, D)** GO and KEGG analysis for evaluating the relationship between the classic cancer pathway and the hub ZWINT gene in LUSC.

**Figure 9:**
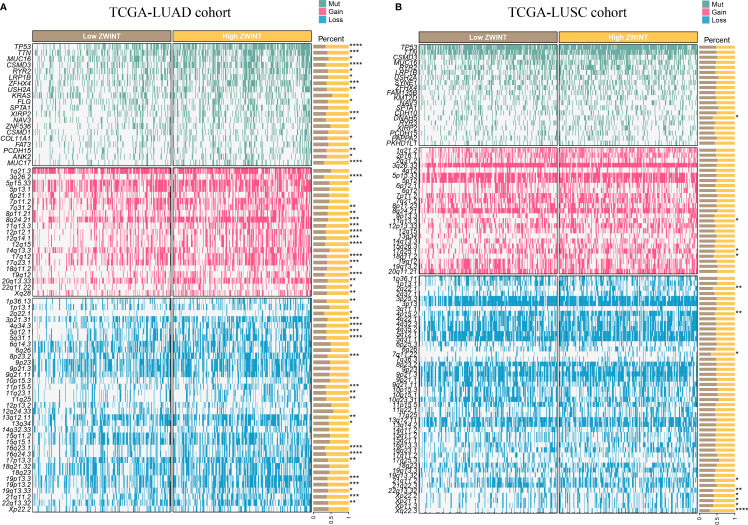
**(A)** Genomic mutation data of LUAD patients with varying ZWINT expression levels. **(B)** Genomic mutation data of LUSC patients with varying ZWINT expression levels. * indicates p < 0.05; ** indicates p < 0.01; *** indicates p < 0.001; and **** indicates p < 0.0001.

GO analysis revealed that the ZWINT gene was involved not only in controlling the cell cycle development in LUAD and LUSC but also in regulating processes such as cell division, chromosomal segregation, nucleoplasm, cell periphery, etc. (**Figures 8A, B**). However, there are still some gaps that need attention. The ZWINT gene has a stronger influence on the development and stability of cell membranes in LUAD and a greater influence on the extracellular matrix and some immunomodulatory responses in LUSC. KEGG analysis results further verified the influence of ZWINT on the advancement of the NSCLC cell cycle (**Figures 8C, D**). In addition, the genomic mutation data of NSCLC patients with varying ZWINT expression levels were also analyzed in this study. As shown in **Figure 9**, the ZWINT gene has a stronger impact on the SNV and CNV mutations in LUAD when compared to those in LUSC. NSCLC patients with different clinical traits demonstrated distinct expression levels of the ZWINT gene (**Supplementary Figure 10**). The male patients with LUAD had considerably higher levels of ZWINT gene expression than the female patients. The expression levels of the ZWINT gene were substantially higher in patients with LUSC younger than or equal to 65 years than in those older than 65 years. For both LUAD and LUSC patients, the expression of the ZWINT gene was significantly associated with the tumor stage. Above all, ZWINT expression was found to be significantly correlated with tumor immune-related pathways, metabolism-related pathways, and cell death-related pathways (**Supplementary Figure 11**). Specifically, ZWINT expression was negatively associated with the activities of the T cell receptor signaling pathway, B cell receptor signaling pathway, toll-like receptor signaling pathway, and cytokine-cytokine receptor interactions (**Supplementary Figure 11A**). A complex regulatory relationship was observed between the ZWINT gene and the classical metabolic pathways of the tumor. As shown in **Supplementary Figure 11B**, a significant positive correlation between the ZWINT gene and pyrimidine metabolism was observed in all types of tumor tissues. However, a different correlation was observed with other metabolic pathways due to tumor heterogeneity. In addition, ZWINT expression was positively related to several cell death pathways in patients with KIRC and THCA (**Supplementary Figure 11C**). ZWINT expression was negatively related to several cell death pathways in patients with CESC, ESCA, and GBM (**Supplementary Figure 11C**).

### 3.5 Expression validation of ZWINT gene in patients with NSCLC

**Figure 10:**
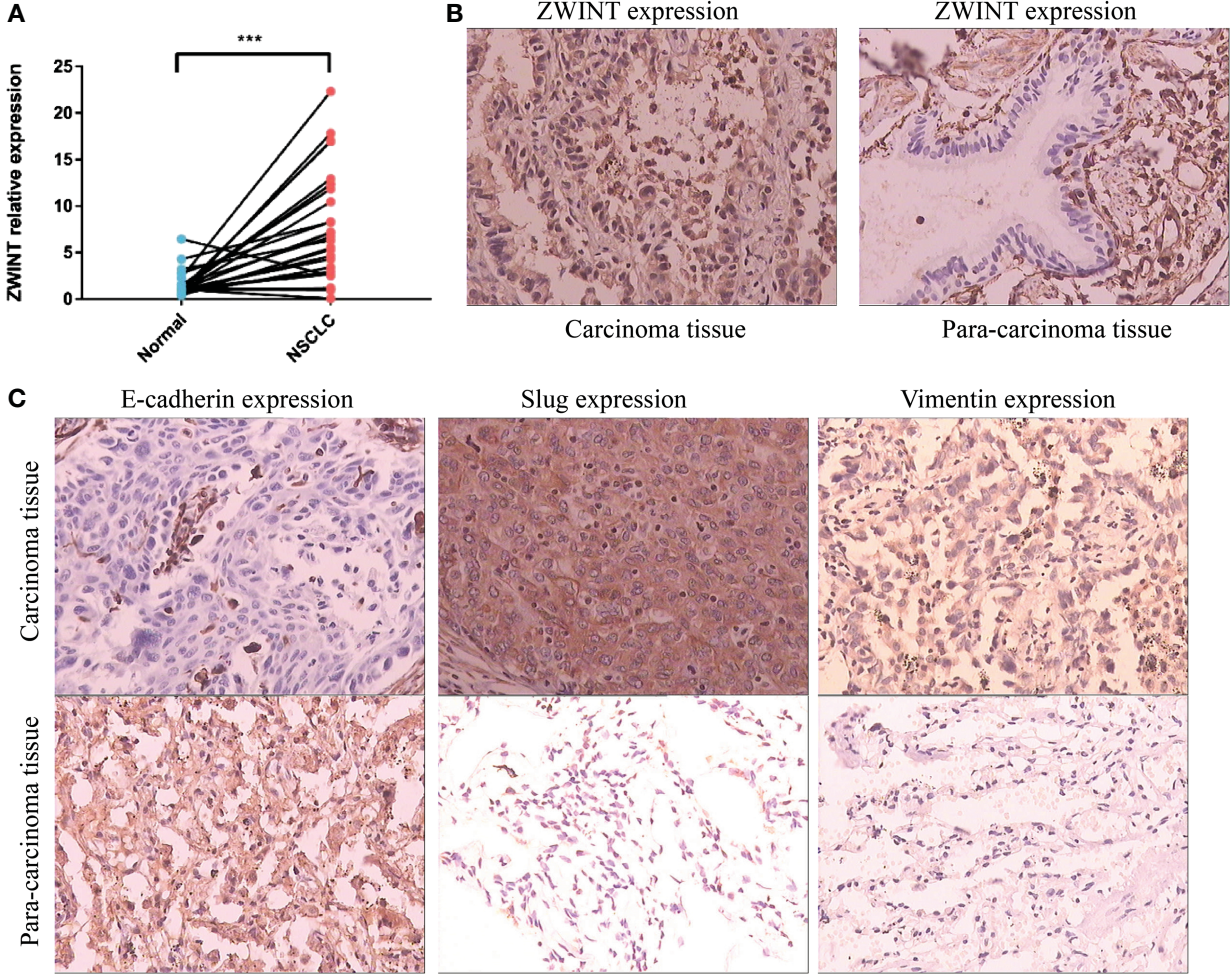
Expression validation of the ZWINT gene in NSCLC. **(A)** RT-qPCR analysis of 30 paired frozen cancerous and paracancerous tissues. **(B)** IHC experiments verified the expression levels of the ZWINT gene in cancerous and paracancerous tissues. **(C)** IHC experiments verified the expression levels of EMT pathway-associated markers in cancerous and paracancerous tissues. *** indicates p < 0.001.

We identified the expression of the ZWINT gene in 30 samples of NSCLC and nearby frozen tissues using RT-qPCR. Consistent with our previous hypothesis, the RT-qPCR data demonstrated that the expression of ZWINT in cancer tissues was much higher than that in the neighboring tissues (**Figure 10A**). The immunohistochemical results further elucidated the up-regulated expression of the ZWINT gene in cancer tissues from the perspective of protein levels (**Figure 10B** and **Table 3**). In addition, the immunohistochemistry studies demonstrated that the expression of E-cadherin was significantly down-regulated in cancerous tissues, while the expression of Vimentin and Slug protein was significantly up-regulated in cancerous tissues (**Figure 10C**). The protein expression levels of ZWINT in NSCLC were negatively linked with E-cadherin, strongly associated with Vimentin, and positively correlated with Slug protein (**Table 4**).

**Table 3:** Protein expression of ZWINT in NSCLC and paracancer tissues (%).

Group	N	Protein expression levels of ZWINT		χ^2^	p
		High(%)	Low(%)		
NSCLC	50	36(72%)	14(28%)	34.313	0.000
Para cancer	50	7(14%)	43(86%)		

Red value means that the p-value is less than 0.05, indicating statistical significance.

**Table 4:** Correlation between ZWINT and E-cadherin, Vimentin, and Slug protein expression (n).

ZWINT	E-cadherin		Vimentin		Slug	
	High	Low	High	Low	High	Low
High	13	22	23	13	26	10
Low	11	4	4	10	5	9
r	-0.332		0.318		0.338	
P	0.020		0.026		0.018	

Red values means that the p-value is less than 0.05, indicating statistical significance.

## 4 Discussion

Cell growth and differentiation are the essential phases of the cell cycle. The control of the cell fate through the cell cycle enables the development and self-renewal of mammalian cells. In other words, signaling pathways involved in the cell cycle regulate cell growth, proliferation, and differentiation. Each of the four phases of the cell cycle, namely, G1, S, G2, and M, occur sequentially and are rigorously controlled. The cell cycle checkpoint is the cell’s feedback control mechanism that decides whether the cell can progress to the subsequent phase. When aberrant events (such as DNA damage) occur, cell cycle checkpoints are involved in halting cell transitions to the next stage, accruing repairs, and promoting the release of a series of repair-functioning proteins. According to tumor research, the formation and progression of tumors are closely connected to the aberrant composition of the control point in the G1/S phase. With the expansion of scientific research, it is generally accepted that cyclin-dependent kinase and cyclin are potential therapeutic targets of anti-tumor medicines. However, it is apparent that the cell cycle is an excellent and intricate network, in which each cell cycle regulator is closely connected, interacts with each other, regulates or inhibits the progression of the entire cell cycle, and ultimately leads to cancer. Therefore, there is an urgent need to use more cutting-edge analytical tools for investigating the possible intermolecular interactions and regulation of gene expression, which can enhance the understanding of the intrinsic characteristics of tumors. Moreover, it may provide a novel alternative therapeutic method.

In this study, 693 cell cycle regulators were identified, among which 93 were differentially expressed in NSCLC and surrounding tissues, and were associated with the clinical stage of the tumor. With the rapid development of the bioinformatics field, people have gradually reached a consensus that differentially expressed genes between cancer and paracancerous tissues are often related to tumorigenesis, while genes related to the clinical stages are often involved in tumor progression. Therefore, the 93 cell cycle regulators identified in this study can be considered to be involved in controlling the incidence and progression of NSCLC to a certain extent, which is of tremendous research significance, and the further bioinformatics analysis and experimental verification in this study were also based on this.

The pan-cancer multi-group properties of these cell cycle regulators are systematically elucidated for the first time in this study, which is one of its novel contributions. The study findings implied that cell cycle signals may have contrasting regulatory effects on different subsets of NSCLC patients since the majority of genes play risk roles in LUAD but protective roles in LUSC. The phenomena have been documented for the first time in this study. Moreover, cell cycle signals are closely associated with other conventional tumor-associated signals, such as metabolic signals, immunological signals, cell death signals, etc. In addition to the consensus clustering of the transcriptome, we conducted a comprehensive analysis of the genomic characteristics of the regulators, such as CNV and SNV.

More importantly, cell cycle signal activity was used for the first time in this study for the effective classification of NSCLC patients into three subgroups. For patients with LUAD, the active cell cycle signal often led to unfavorable clinical results. However, for patients with LUSC, the longer their survival time, the more active the cell cycle signal. Consistent with our previous pan-cancer analysis findings, it was observed that there is evident molecular heterogeneity in patients with NSCLC, the reason for heterogeneity may be related to its pathological type, and cell cycle signaling, as the most fundamental process in cell survival, plays nearly opposite roles in LUAD and LUSC. In NSCLC patients with distinct cell cycle activity, the immunological microenvironment, metabolic reprogramming, and cell death mechanisms are often distinct. This further validates the connection between these complex networks and promotes the progression and development of malignancies.

ZWINT is a protein that interacts with ZW10 and is encoded by the ZWINT gene. This protein is essential for chromosomal mobility and spindle checkpoint regulation, as well as mitosis and cell proliferation (4, 31). It is generally accepted that mitotic abnormalities are characteristic of a majority of malignant tumors. Although the precise function of the molecular composition of the centromere and the interactions between various components of the centromere are unknown, there is growing evidence that ZWINT is frequently over-expressed in several tumors and associated with poor clinical prognosis and early recurrence (32). ZWINT has been demonstrated to diminish chromosomal stability during the development of cancer, indicating that it may function as an oncoprotein (33). The high expression of ZWINT is strongly associated with tumor recurrence, which is a possible risk factor for the high recurrence rate and poor survival rate in patients with liver cancer (34). Endo et al. revealed that the high expression of ZWINT is associated with the overall poor survival rate of LUAD, and ZWINT has a high sensitivity for early screening of lung cancer (35). Mou et al. have shown that ZWINT may affect the proliferation and migration of melanoma cells by regulating the expression of c-Myc (36). Kim et al. observed that ZWINT is abundantly expressed in pan-cancer cells and tissues and enhances pan-cancer cell proliferation and invasion through NF-kB signal transduction (37).

PCR and immunohistochemistry results indicated that the expression levels of the ZWINT gene were considerably higher in NSCLC cancer tissues than that in the surrounding tissues, and the ZWINT gene may contribute to disease progression by increasing the epithelial-mesenchymal transition (EMT). E-cadherin (cadherin), which is completely expressed on the membrane surface of epithelial cells, is the primary hallmark of EMT epithelioid cells, and a decline in the E-cadherin expression reduces the adhesion between cells (38). Vimentin and Slug are the primary markers of EMT interstitial-like cells (39). Slug overexpression may activate ERK2 in the nucleus, decrease E-cadherin production, and enhance the incidence of EMT (40, 41). In this study, ZWINT protein expression in NSCLC was negatively connected with E-cadherin and strongly correlated with Vimentin and Slug proteins. It is hypothesized that elevated ZWINT expression may upregulate the Vimentin and Slug proteins and downregulate the E-cadherin protein, thereby promoting the occurrence of EMT. This plays a significant role in the metastasis and development of NSCLC.

## 5 Conclusions

This study comprehensively characterized the pan-cancer cell cycle regulatory landscape for the first time. We successfully identified the molecular heterogeneity in patients with NSCLC based on the cell cycle activities. ZWINT has been proven to be significantly up-regulated in NSCLC tissues compared to paracancerous tissues, which might promote the progression of tumors through activation of the EMT pathway.

## Data availability statement

The original contributions presented in the study are included in the article/**Supplementary Material**. Further inquiries can be directed to the corresponding author.

## Ethics statement

The studies involving human participants were reviewed and approved by The Ethics Committee of the First Affiliated Hospital of Dalian Medical University. The patients/participants provided their written informed consent to participate in this study.

## Author contributions

All the authors bear full responsibility for the content of this manuscript. All the authors were involved in the conception of this study, data gathering and analysis, manuscript drafting, and manuscript revision. All authors contributed to the article and approved the submitted version.

## Supplementary material

Supplementary Figure 1 Venn diagram highlighting the shared differentially expressed cell cycle-related genes in both LUAD and LUSC cohorts.

Supplementary Figure 2 mRNA expression levels of ZWINT in different types of cancers **(A)** ZWINT expression traits in cancerous and normal samples based on the TCGA cohort. **(B)** ZWINT expression traits in cancerous and normal samples based on the TCGA and GTEx cohorts.

Supplementary Figure 3 Prognostic significance of ZWINT gene in pan cancer by univariate COX regression analysis.

Supplementary Figure 4 Overall survival of the ZWINT gene in pan cancer by KM analysis.

Supplementary Figure 5 Progression-free interval of ZWINT gene in pan cancer by KM analysis.

Supplementary Figure 6 Disease-specific survival of ZWINT gene in pan cancer by KM analysis.

Supplementary Figure 7 Disease-free interval of ZWINT gene in pan cancer by KM analysis.

Supplementary Figure 8 Relationship between the expression levels of ZWINT and immune checkpoints.

Supplementary Figure 9 Relationship between ZWINT and immunotherapy.

Supplementary Figure 10 Association of ZWINT expression with clinical characteristics of NSCLC patients.

Supplementary Figure 11 Association of ZWINT expression with **(A)** immune pathways, **(B)** metabolic pathways, and **(C)** cell death pathways in pan cancer.
