# Supplementary figures and images for: Identification of the cell cycle characteristics of non-small cell lung cancer and its relationship with tumor immune microenvironment, cell death pathways, and metabolic reprogramming
Source: Front Endocrinol (Lausanne). 2023 Apr 6;14:1147366. doi: 10.3389/fendo.2023.1147366 (PMC10117961; doi:10.3389/fendo.2023.1147366)

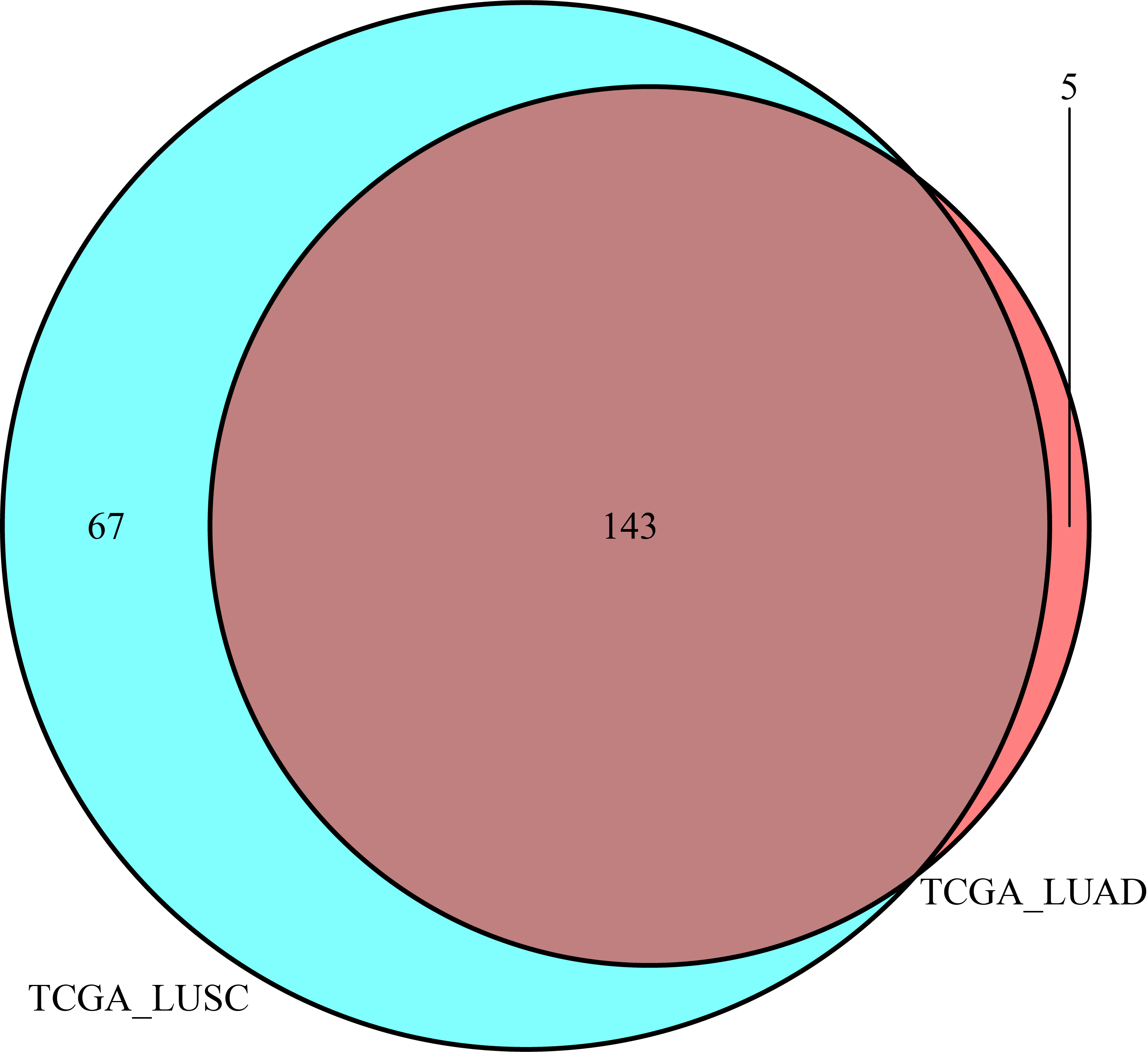

Supplement: Supplementary Figure 1 — Venn diagram highlighting the shared differentially expressed cell cycle-related genes in both LUAD and LUSC cohorts. [file Image_1.jpeg]

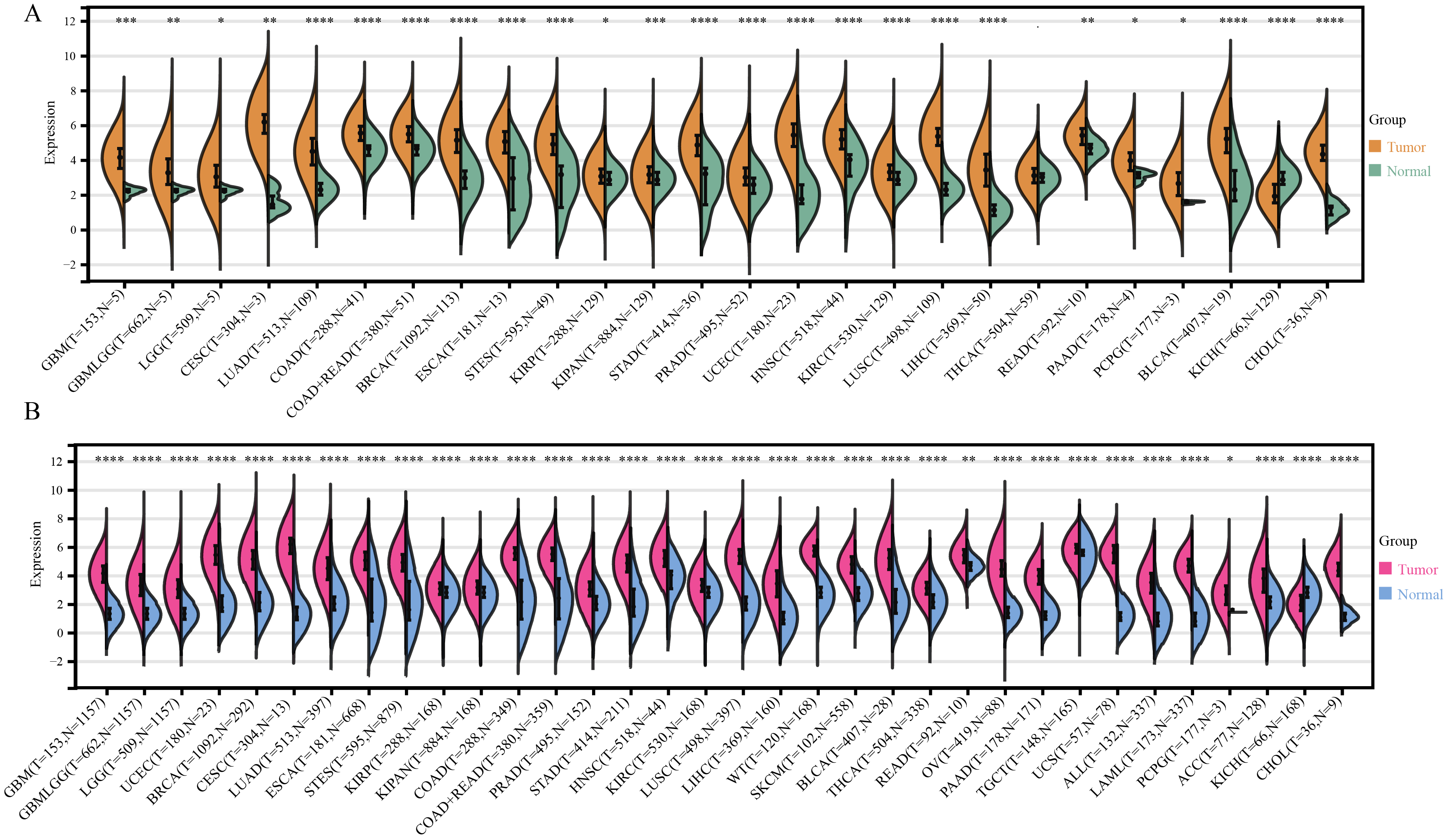

Supplement: Supplementary Figure 2 — mRNA expression levels of ZWINT in different types of cancers (A) ZWINT expression traits in cancerous and normal samples based on the TCGA cohort. (B) ZWINT expression traits in cancerous and normal samples based on the TCGA and GTEx cohorts. [file Image_2.jpeg]

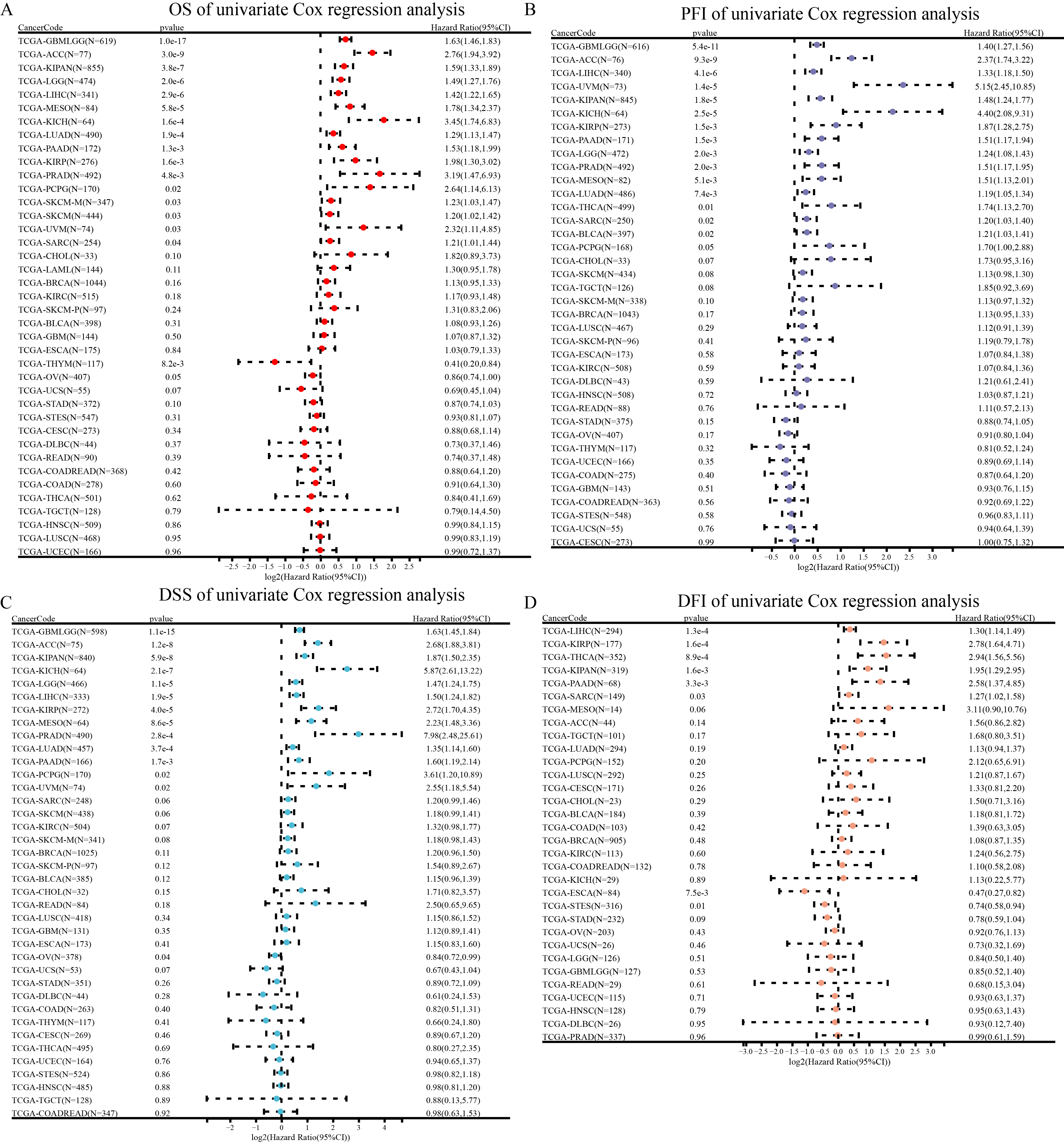

Supplement: Supplementary Figure 3 — Prognostic significance of ZWINT gene in pan cancer by univariate COX regression analysis. [file Image_3.jpeg]

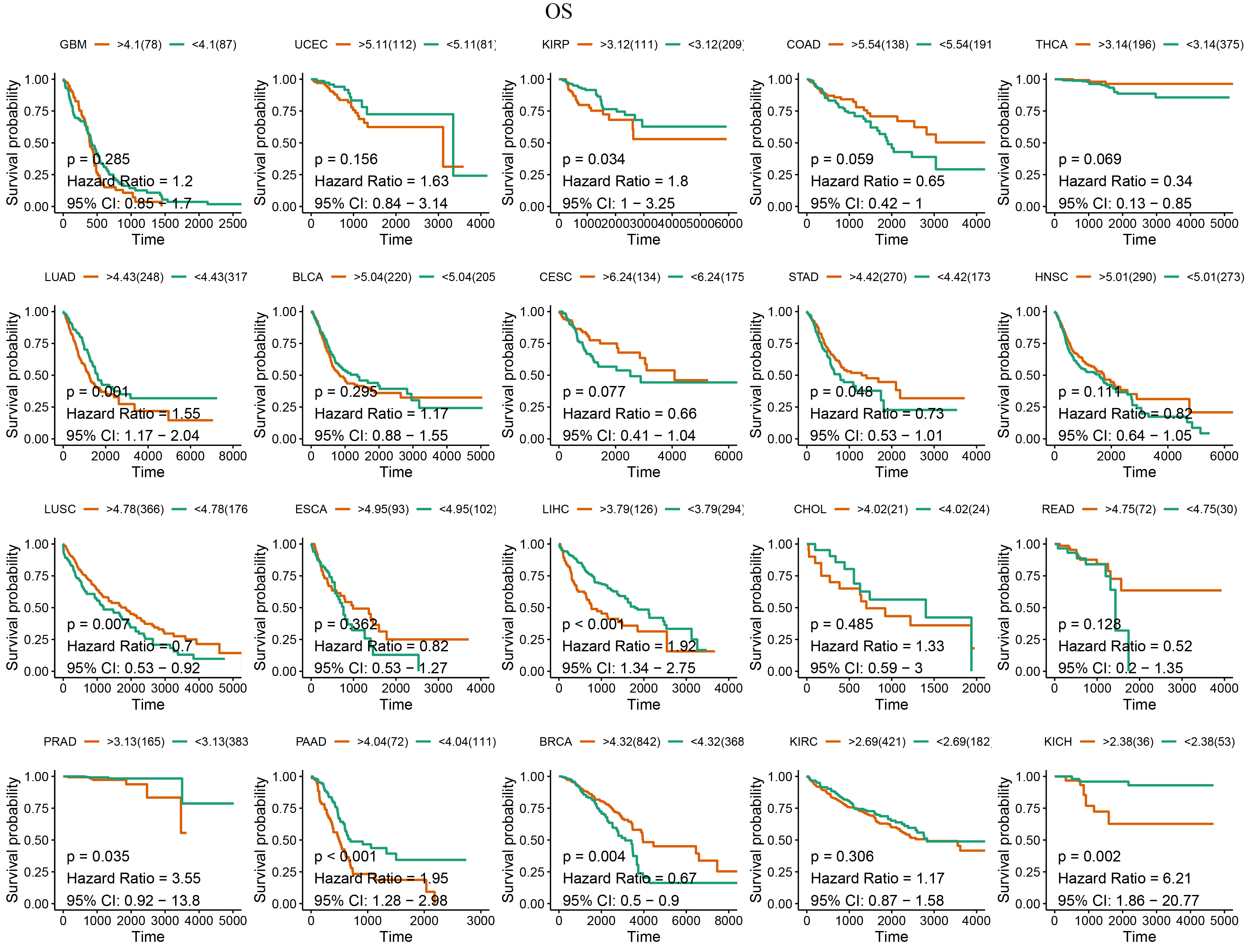

Supplement: Supplementary Figure 4 — Overall survival of the ZWINT gene in pan cancer by KM analysis. [file Image_4.jpeg]

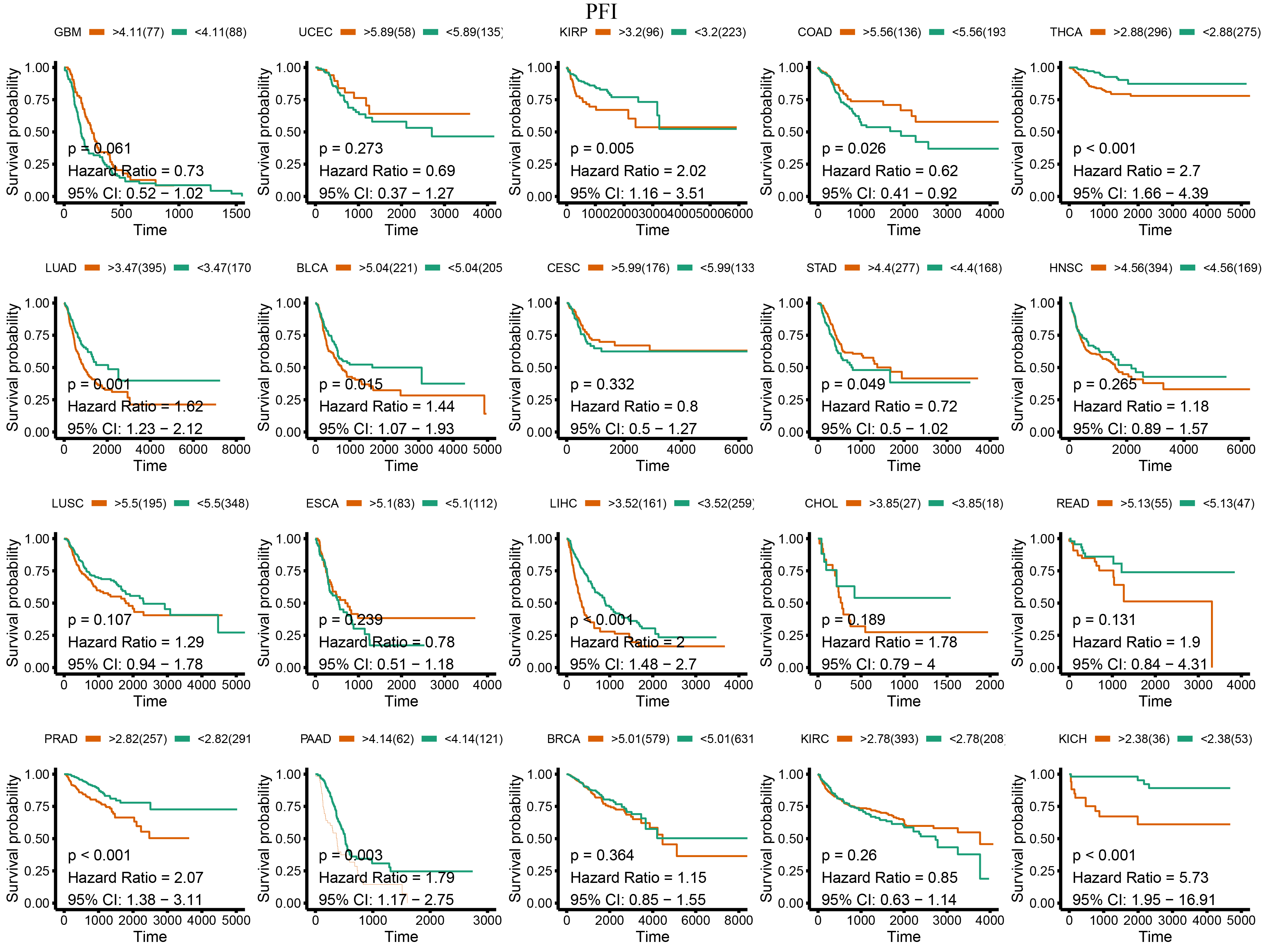

Supplement: Supplementary Figure 5 — Progression-free interval of ZWINT gene in pan cancer by KM analysis. [file Image_5.jpeg]

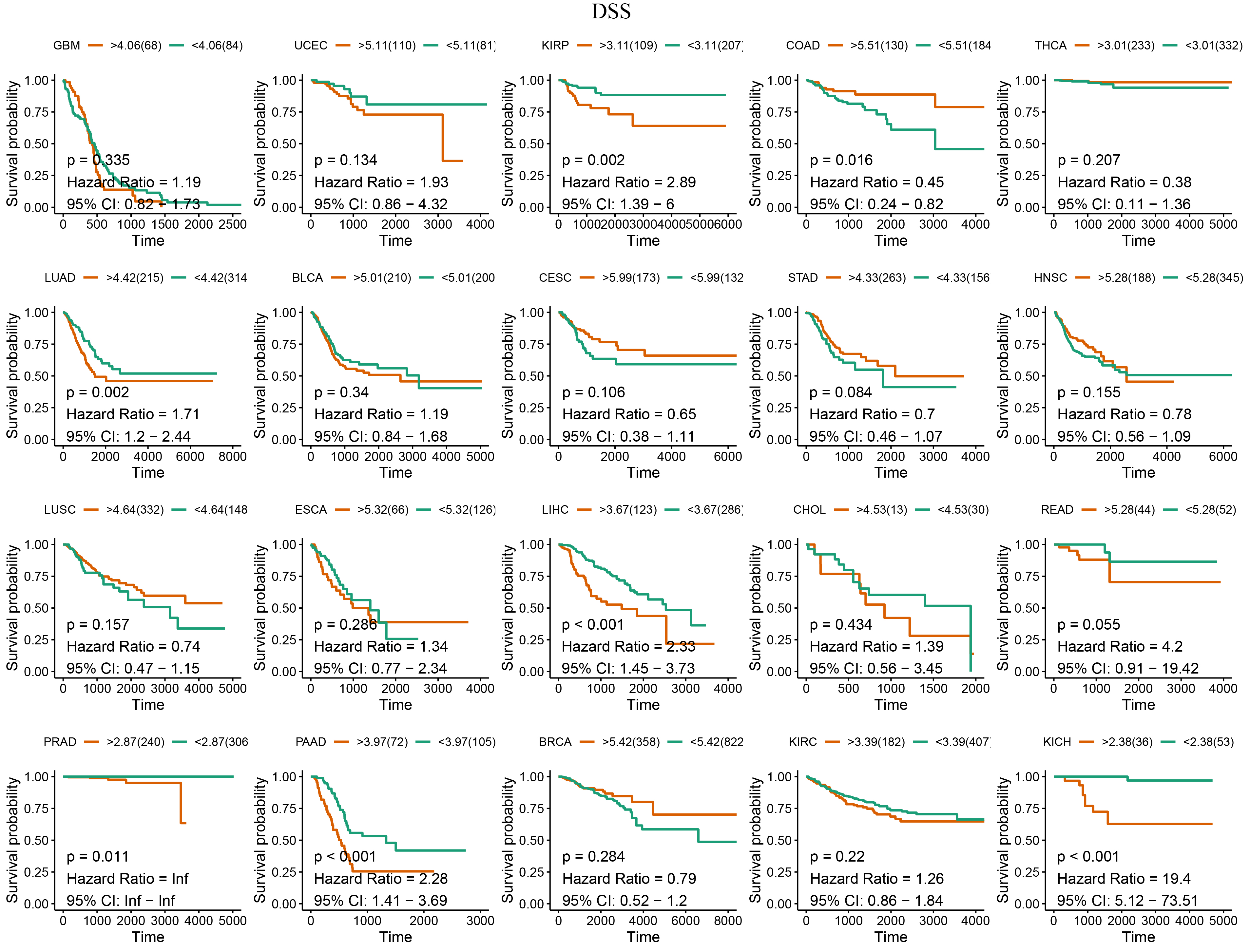

Supplement: Supplementary Figure 6 — Disease-specific survival of ZWINT gene in pan cancer by KM analysis. [file Image_6.jpeg]

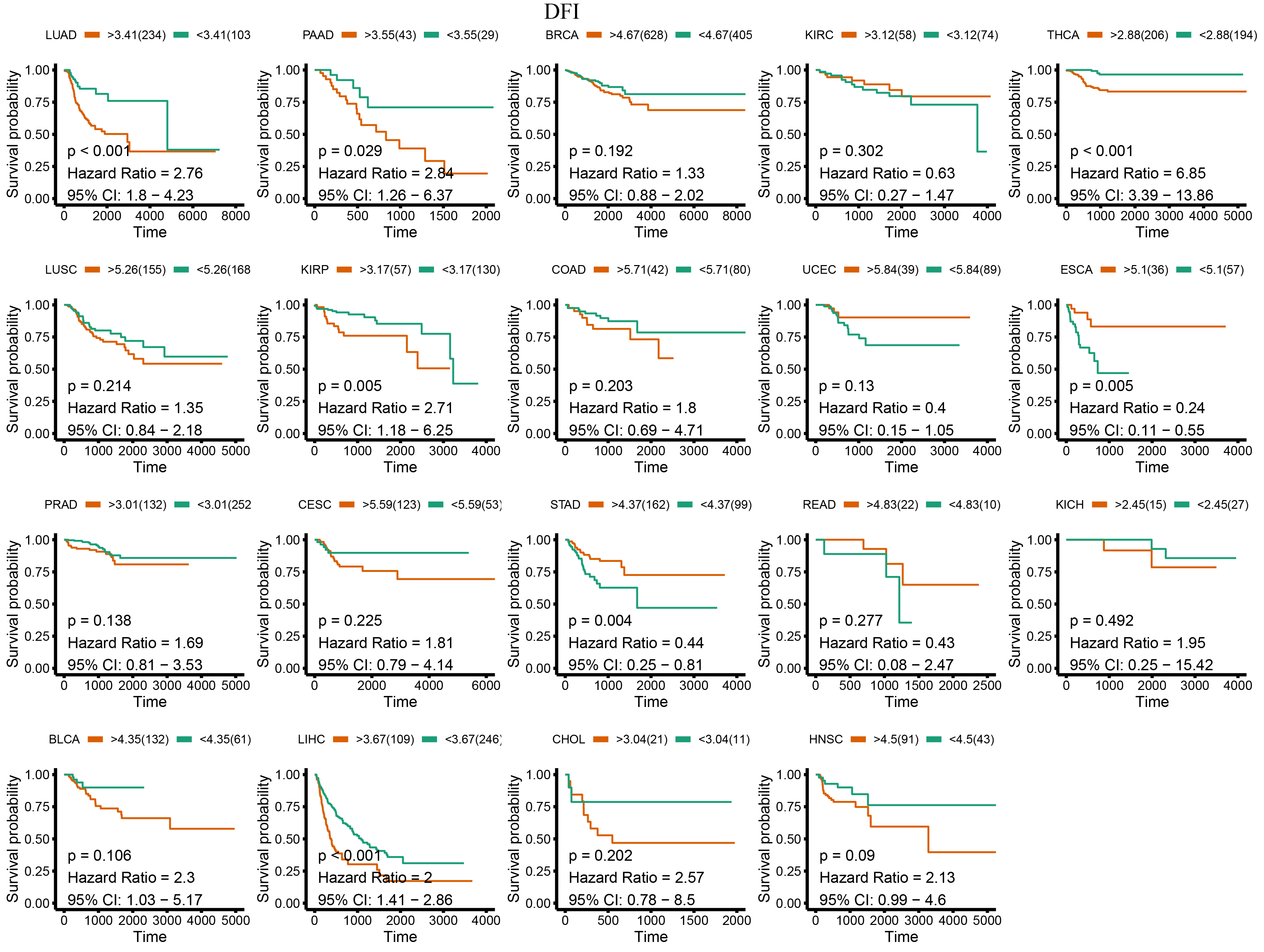

Supplement: Supplementary Figure 7 — Disease-free interval of ZWINT gene in pan cancer by KM analysis. [file Image_7.jpeg]

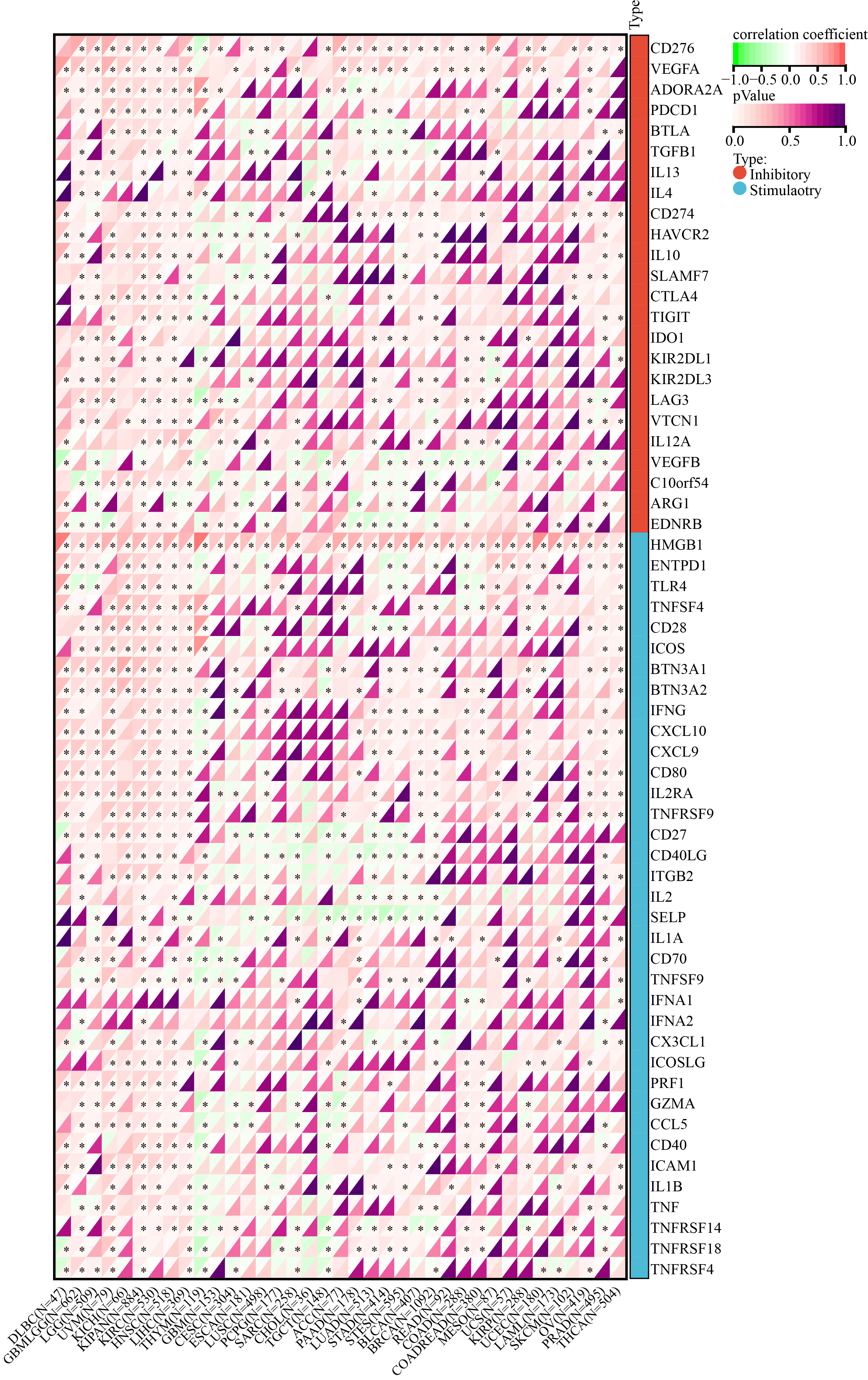

Supplement: Supplementary Figure 8 — Relationship between the expression levels of ZWINT and immune checkpoints. [file Image_8.jpeg]

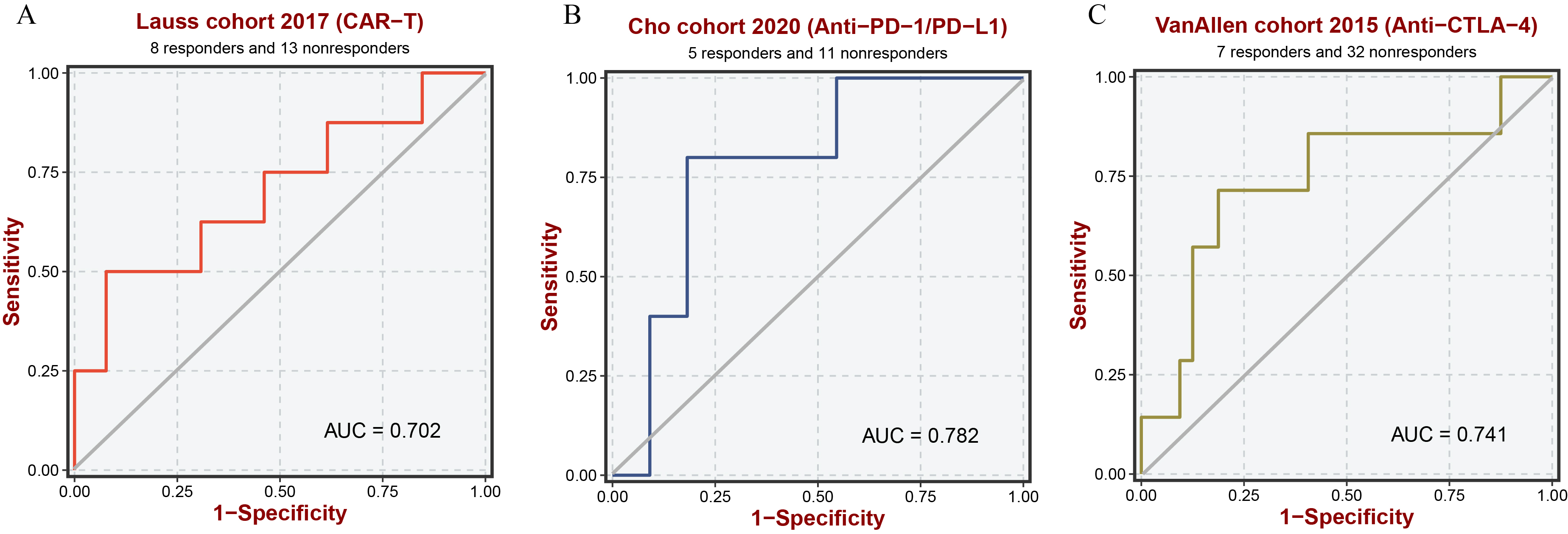

Supplement: Supplementary Figure 9 — Relationship between ZWINT and immunotherapy. [file Image_9.jpeg]

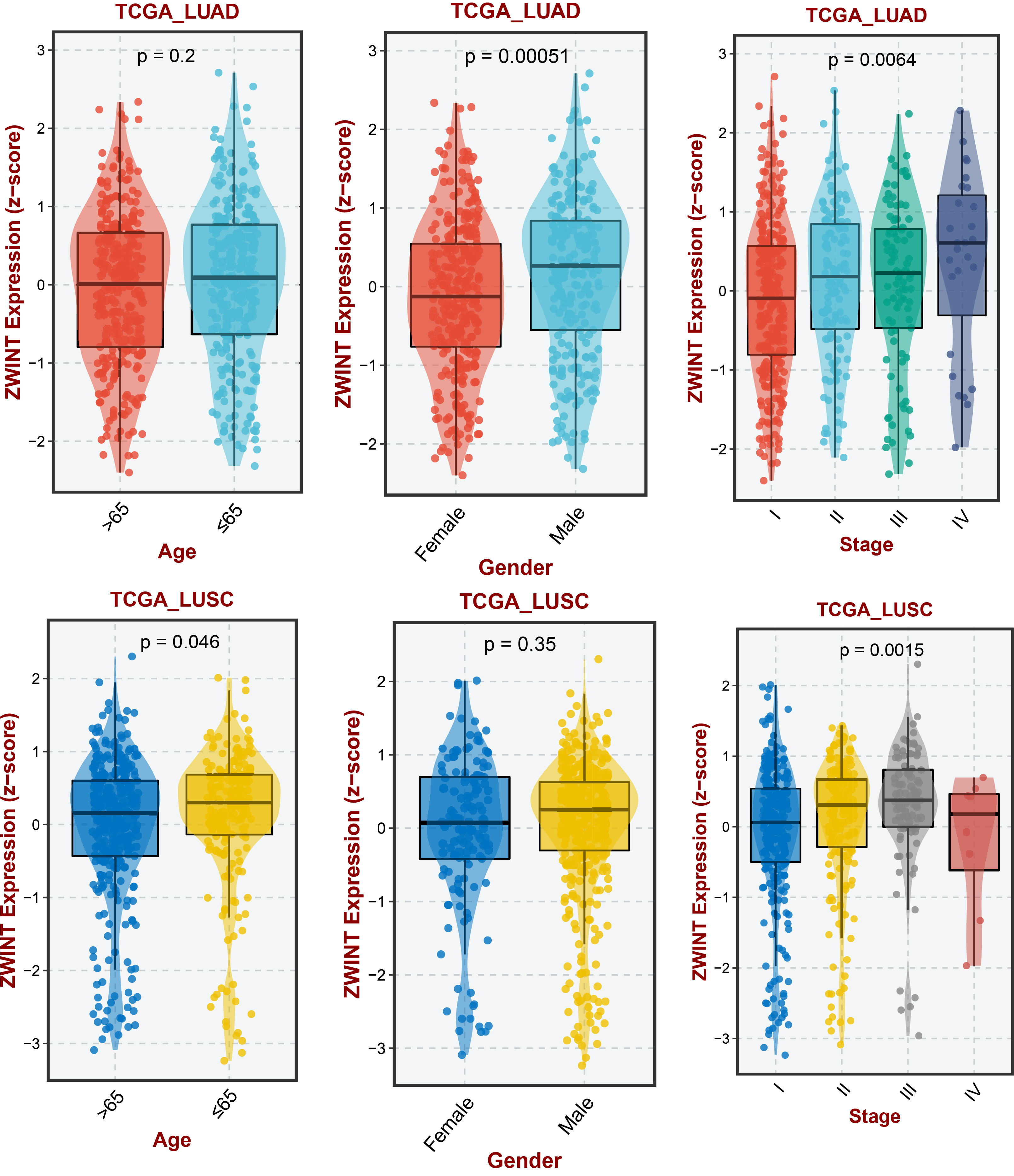

Supplement: Supplementary Figure 10 — Association of ZWINT expression with clinical characteristics of NSCLC patients. [file Image_10.jpeg]

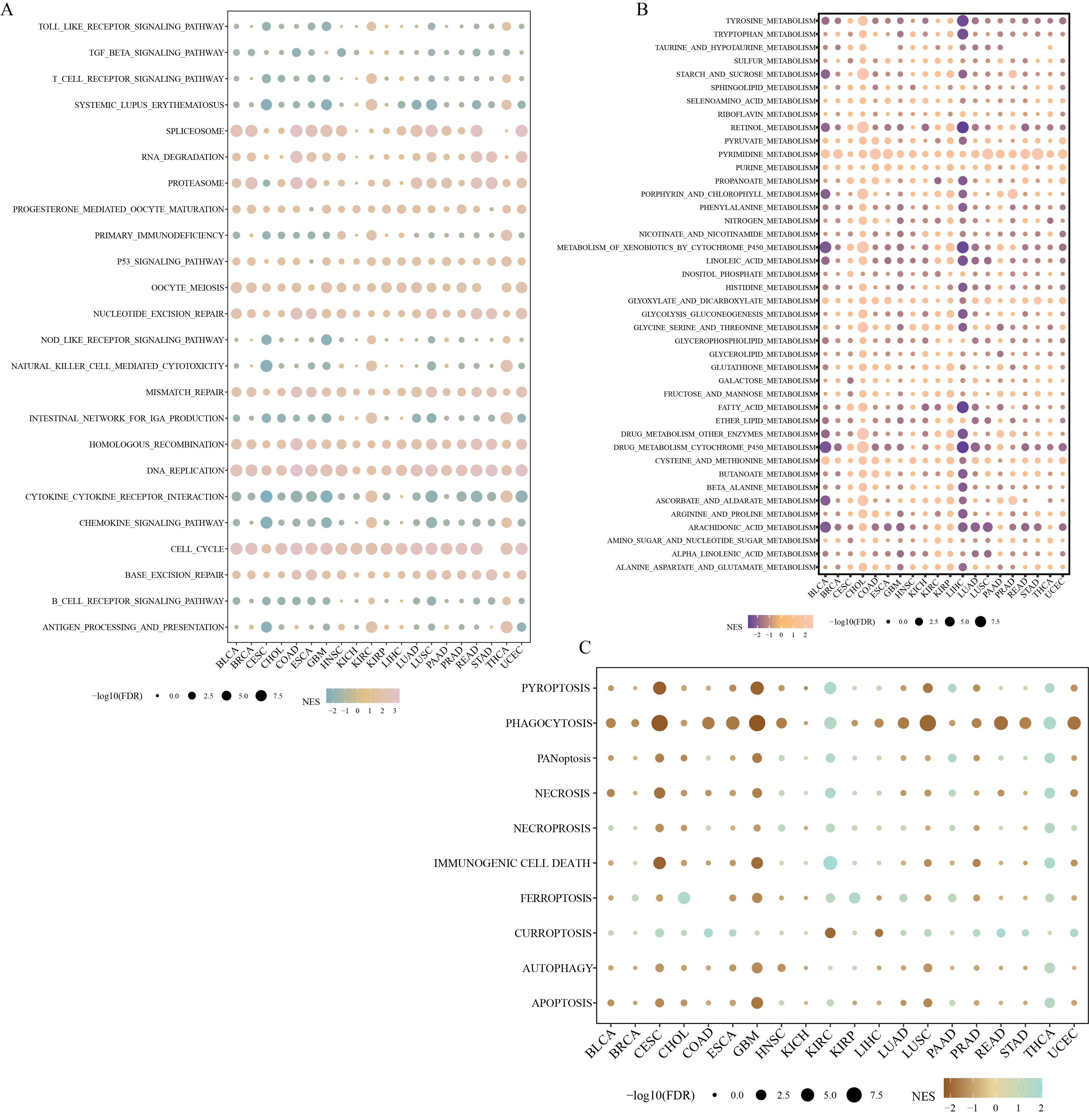

Supplement: Supplementary Figure 11 — Association of ZWINT expression with (A) immune pathways, (B) metabolic pathways, and (C) cell death pathways in pan cancer. [file Image_11.jpeg]
